# Auxin Biosynthesis, Transport, Signaling, and Its Roles in Plant Leaf Morphogenesis

**DOI:** 10.3390/plants15010072

**Published:** 2025-12-25

**Authors:** Han Zheng, Qian Zhang, Qun Liu, Jingjuan Li, Yihui Zhang, Lixia Wang, Jianwei Gao

**Affiliations:** 1 Shandong Key Laboratory of Bulk Open-Field Vegetable Breeding, Ministry of Agriculture and Rural Affairs Key Laboratory of Huang Huai Protected Horticulture Engineering, Institute of Vegetables, Shandong Academy of Agricultural Sciences, Jinan 250100, China; zhenghan1216@163.com (H.Z.); lijj0620@163.com (J.L.); zyh_0923@163.com (Y.Z.); 13148310672@163.com (L.W.); 2 College of Life Science, Shandong Normal University, Jinan 250100, China; 13295418863@163.com (Q.Z.); 19811827686@163.com (Q.L.)

**Keywords:** auxin, biosynthesis, leaf morphogenesis, signaling, transport

## Abstract

Leaf morphogenesis is governed by a tightly integrated regulatory network centered on auxin, which operates through a sequential axis of synthesis, transport, and signal transduction. This review elucidates how pivotal molecular hubs previously identified in this regulatory network, including biosynthetic enzymes, polar transporters, and auxin response factors, interconnect through dynamic feedback mechanisms to orchestrate leaf initiation, polarity establishment, and the determination of its final size and shape. Notably, recent breakthroughs are transforming the field: the re-evaluation of established pathways like indole-3-acetaldoxime (IAOx), whose direct contribution to auxin pools is under scrutiny, hinting at the existence of undiscovered enzymes or alternative metabolic branches and the paradigm-shifting discovery that cAMP functions as a second messenger produced by Transport Inhibitor Resistant 1/Auxin signaling F-box (TIR1/AFB) receptors, which directly activates *Auxin Response Factor* (*ARF*)-mediated transcription. These foundational mechanistic insights provide the critical groundwork for application. Key network nodes—such as PIN-FORMED (PIN) transporters and YUCCA (YUC) flavin-containing monooxygenases—are now validated targets for crop improvement. Consequently, the elucidated network serves as a blueprint for rationally designing crop architecture.

## 1. Introduction

Auxin, one of the most extensively studied phytohormones, was first isolated as indole-3-acetic acid (IAA), which is recognized as the primary bioactive auxin in plants. Its identification emerged from investigations into light- and gravity-induced tropic responses [1]. In addition to IAA, plants synthesize various other endogenous auxins, including indole-3-butyric acid (IBA), phenylacetic acid (PAA), and 4-chloroindole-3-acetic acid (4-Cl-IAA). Studies in *Arabidopsis* have demonstrated that the enzymatic activity of anthranilate synthase (ASA1/ASB1) is essential for IBA-to-IAA conversion during adventitious root formation [2]. Nevertheless, whether IBA also functions independently as an auxin per se remains an open question that warrants further investigation. Similarly, the functions of PAA and 4-Cl-IAA have yet to be fully elucidated.

Auxin biosynthesis occurs through both tryptophan (TRP)-dependent and TRP-independent pathways. The TRP-dependent auxin biosynthesis process encompasses various parallel pathways that converge at the production of IAA, with IAOx, indole-3-acetamide (IAM), and indole-3-pyruvic acid (IPyA) being the most prevalent intermediates [3]. The TRP-independent pathway was proposed following the discovery of mutants with defects in TRP biosynthesis in maize and *Arabidopsis*, which retained their capability to produce IAA [4]. Indole-3-glycerol phosphate (IGP) may serve as a crucial intermediate in TRP-independent auxin biosynthesis due to its conversion into indole by indole synthase (INS) and its paralog α-tryptophan synthase (TSA) [5,6]. However, further investigations are necessary to characterize this pathway and identify the proteins involved in signal transduction.

Once synthesized, the distribution of auxin is dynamically regulated within tissues. It traverses through the phloem and, more critically, is transported via polar auxin transport (PAT), which establishes directional concentration gradients that guide developmental patterning [7]. PAT plays a vital role in the short-distance distribution of auxin, which depends on membrane-localized transporters. This process involves AUXIN RESISTANT1/LIKE AUX (AUX1/LAX) for influx and PIN as well as ABC-B/MULTIDRUG RESISTANCE/P-GLYCOPROTEIN (ABCB/MDR/PGP) proteins for efflux [8].

As a polarized signaling molecule, auxin connects the polarity of individual cells with that of tissues and organs by regulating the polar localization of PIN proteins. Research has confirmed that TIR1/AFBs mediate auxin channelization by indirectly influencing the phosphorylation of PINs [9,10]. Additionally, Transmembrane Kinases (TMK) proteins can directly interact with PIN1 and PIN2, respectively, regulating their localization to affect channelization [11]. Recently, the AUXIN-BINDING PROTEIN1 (ABP1)/ABP1-LIKE 3 (ABL3)—TMK1 receptor module has been identified as a regulator of PIN2-mediated auxin fluxes in root gravitropism [12]. These findings highlight that the interplay between cell-surface auxin signaling and PIN-mediated polar auxin transport is presumably essential for self-organizing developmental processes.

Auxin orchestrates nearly all aspects of plant growth, from morphogenesis and organ patterning to tropic responses [13]. In leaves, it promotes initiation, establishes polarity, and regulates the final dimensions as well as morphological development [14]. In leaf development initiation, PIN1 transports auxin to the leaf primordium’s initial site, while the AUX1/LAX family stabilizes PIN1-mediated auxin patterning, regulating leaf primordia localization and initiation [15,16]. In leaf polarity establishment, the *ARF* transcription factor family, essential for auxin signaling, is involved in establishing all three axes: the adaxial-abaxial axis, the proximal-distal axis, and the medio-lateral axis [17,18,19,20]. The *WUSCHEL-like homeobox* (*WOX*) family plays a key role in establishing leaf polarity [21,22]. In the leaf margin, *WOXs* regulate *YUCCA* expression, an essential enzyme for auxin synthesis [23]. Thus, the WOX-YUCCA-auxin regulatory module coordinates growth along the proximo-distal and medio-lateral axes, shaping leaf form. In regulating leaf morphology, the CUP-SHAPED COTYLEDON2 (CUC2)-PIN1-auxin genetic module plays a crucial role [24,25]. The transcription factor *ARF2* regulates the cell cycle progression to control cell proliferation, which is a critical determinant of leaf size in plants [26]. These outcomes indicate that auxin biosynthesis, transport, and signaling converge into a significantly interconnected regulatory network that precisely orchestrates leaf morphogenesis with both accuracy and adaptability. Disentangling the molecular architecture, dynamic interactions, and functional rationale of this network is pivotal for the rational engineering of crop architecture and performance in response to changing environmental conditions.

Although the roles of auxin in plant development have been widely reviewed, a stage-resolved and mechanistic synthesis specifically focused on leaf morphogenesis remains limited. To bridge this gap, we propose a comprehensive framework that integrates auxin biosynthesis, polar transport, signal transduction, and morphogenetic output into a unified developmental cascade. By deconstructing leaf morphogenesis into three phases (initiation, polarity establishment, and final form determination), we map key regulatory nodes to each stage, providing a systematic view of auxin-mediated leaf development. However, the principal nodes orchestrating its dynamic regulation remain incompletely understood. Future efforts should thus deepen understanding of these regulatory cores, which will be essential for constructing a more integrated model of auxin-directed development. Such insight will in turn inform the rational design of crop architecture, offering strategic pathways toward enhanced agricultural productivity and sustainability.

## 2. Types of Auxins and Their Functions in Plants

**Figure 1:**
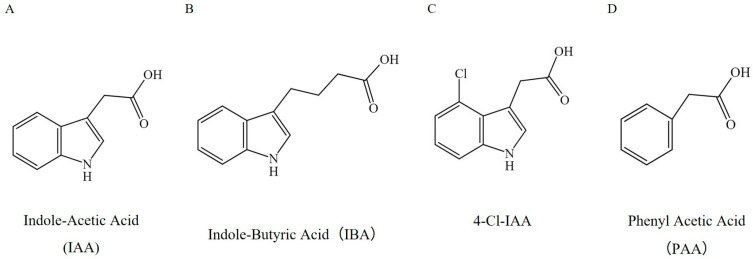
The chemical structures of the four natural auxins in plants. Auxins share similar chemical structures, all featuring a heterocyclic aromatic ring with a bicyclic structure, which comprises a benzene ring fused to a pyrrole ring, along with a carboxyl group.

Auxins represent a class of endogenous plant hormones, among which IAA is recognized as the most prevalent and biologically active form found in nature. Structurally, IAA features an indole ring connected to an acetic acid side chain, forming a fundamental scaffold that contributes significantly to the structural diversity within the auxin family [27]. While IAA, along with indole-3-butyric acid (IBA), 4-chloroindole-3-acetic acid (4-Cl-IAA), and phenylacetic acid (PAA) are the primary endogenous auxins present in plants—each differing in its aromatic system or side-chain structure—they all share characteristic auxin activity that regulates a variety of developmental processes [8,28] (Figure 1). These processes encompass cell division and expansion, embryogenesis, organogenesis, and adaptation to environmental stimuli, thereby highlighting the pivotal role of auxins in driving plant growth and signaling mechanisms.

IBA is the second auxin identified, following IAA, and its chemical structure closely resembles that of IAA, differing only by the presence of two additional methylene groups on the side chain. This structural discrepancy may cause IBA to align its cyclic system horizontally rather than perpendicularly to the base of the TIR1-Aux/IAA co-receptor pocket, ultimately hindering IBA’s ability to bind to the TIR1 receptor [29]. Genetic evidence indicates that the activity of IBA derives from its conversion into IAA in several plant species, including *Arabidopsis*, hazelnut, and elm [30,31]. Notably, IBA has been linked with adventitious root formation and demonstrates greater effectiveness than IAA due to its stability in both solutions and cellular tissues [32]. The impact of IBA on root development has also been reported in *Arabidopsis* as well as various economically important crops such as tomato, *Camellia sinensis*, *Fagopyrum tataricum*, *Mangifera indica*, *Olea europaea*, *Phytolacca americana* L., and Prunus [33,34,35,36,37,38,39,40].

In recent years, 4-Cl-IAA has been identified in plants such as *Medicago*, *Melilotus*, and *Trifolium*, thereby challenging the previously established notion that this compound was confined exclusively to the *Fabaceae* family [41]. The concentrations of both IAA and IBA were found to be elevated in the ovaries of oat containing developing embryos when compared to those lacking such embryos. Notably, 4-Cl-IAA was detected solely in ovaries devoid of developing embryos [2]. Furthermore, 4-Cl-IAA can mimic seed activity by stimulating growth in the seedless pea pericarp through mechanisms that involve inducing gibberellin (GA) biosynthesis, inhibiting ethylene (ETH) effects, and directly promoting auxin-mediated growth of the pea pericarp [42]. The application of 4-Cl-IAA resulted in a prolonged inhibition of *PsAFB6*, *PsTIR1b*, and *PsAFB4* expression within deseeded pea pericarps. In contrast, the administration of IAA did not significantly alter the expression levels of various auxin receptors including *PsTIR1a*, *PsTIR1b*, *PsAFB2*, and *PsAFB4* [42,43].

PAA, an auxin derived from phenylalanine, was recognized for its auxin activity nearly a century ago and is commonly found in various plants. Treatment of *Arabidopsis* seedlings with PAA has been shown to enhance the formation of lateral roots, even though PAA exhibits an activity that is 10- to 20-fold lower than that of IAA [44]. The homeostasis between IAA and PAA has been demonstrated to be regulated through modulatory processes involving auxin conjugation. Furthermore, the accumulation of PAA triggers the expression of genes associated with tryptophan and IAA biosynthesis, indicating a complex regulatory network governing auxin homeostasis [45]. Nevertheless, the precise mechanisms underlying the actions of these hormones remain largely unknown.

## 3. Auxin Biosynthesis Pathways

**Figure 2:**
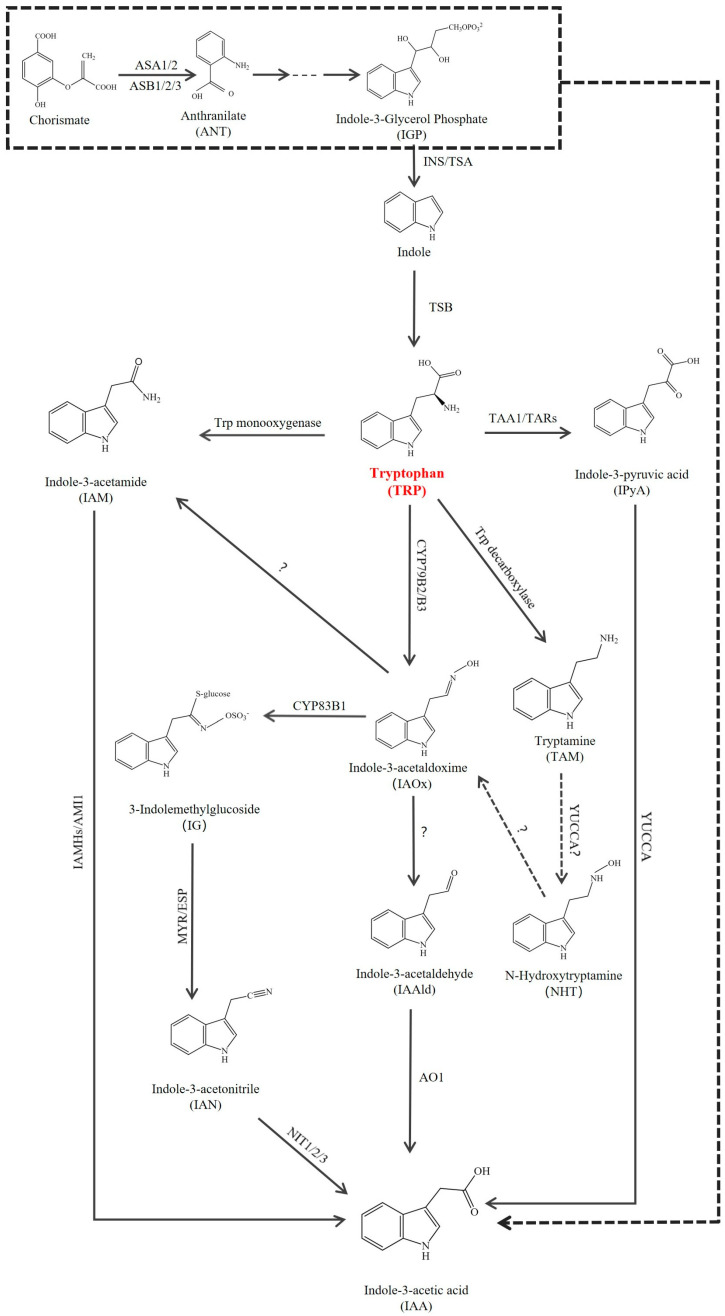
Potential pathways for IAA biosynthesis in *Arabidopsis*. The de novo biosynthetic pathway of IAA initiates with Trp (marked in red) or its precursors. The enzymes identified in *Arabidopsis* are indicated on the arrows, while the conversion processes with unidentified genes are marked with question marks. Dashed lines represent unclear synthetic routes. Abbreviations: Indole-3-acetamide amidohydrolase (AMI), Anthranilate (ANT), Aldehyde oxidase 1 (AO1), Anthranilate synthase α (ASA), Anthranilate synthase β (ASB), Cytochrome P450 (CYP79B2, CYP79B3, CYP83B3), Epithiospecifier protein (ESP), Indole-3-acetaldehyde (IAAld), Indole-3-acetamide (IAM), Indole-3-acetamide hydrolase (IAMH), Indole-3-acetonitrile (IAN), Indole-3-acetaldoxime (IAOx), 3-Indolemethylglucoside (IG), Indole-3-Glycerol Phosphate (IGP), Indole synthase (INS), Indole-3-pyruvic acid (IPyA), Myrosinase (MYR), N-Hydroxytryptamine (NHT), Nitrilase (NIT), Tryptophan aminotransferase of *Arabidopsis* (TAA), Tryptophan Aminotransferase-Related (TAR), Tryptamine (TAM), Tryptophan synthase α (TSA), Tryptophan synthase β (TSB).

Extensive research has been undertaken to clarify the biosynthesis of auxins, particularly IAA. The biosynthesis of IAA occurs through multiple pathways, encompassing both tryptophan-independent and tryptophan-dependent mechanisms, with Indole acting as the pivotal branching point (Figure 2).

The TRP-independent pathway was initially proposed based on molecular analyses of the orange pericarp mutant (*orp*) in maize and the *trp* mutants in *Arabidopsis*. Both of these mutants exhibit defects in TRP synthesis but demonstrate significantly elevated levels of IAA compared to their wild-type counterparts [4,46]. In plants, the biosynthesis of TRP within the chloroplast is initiated by ASA and ASB converting chorismate into anthranilate (ANT) [47]. It was proposed that IGP serves as a vital intermediate product in the TRP-independent auxin biosynthesis pathway. Both INS and TSA possess the capability to catalyze the transformation of IGP into Indole [5,6,48]. Ultimately, Indole is transformed into TRP by the TSB enzyme [49]. However, our comprehension of the gene regulatory mechanisms and the specifics of enzymatic reactions within TRP-independent pathways remains markedly limited. Consequently, further research is essential to elucidate various steps in this pathway and to identify the proteins involved, as these proteins may substantially influence plant growth and development.

The TRP-dependent pathway comprises four alternative routes mediated by transamination enzymes and decarboxylation processes, which include the IPyA, IAOx, IAM, and TAM sub-routes. Several genes involved in the TRP-dependent pathway of auxin biosynthesis in *Arabidopsis* have been identified (Table 1). Among these key genes, four are primarily linked to alterations in leaf size (*TSB*, *TAA/TAR*, *CYP79B2/B3* and *YUCCA*), two are associated with cotyledon morphology (*CYP83B1* and *AO1*), and one is related to leaf rolling (*YUCCA*). This distribution underscores the specific roles that distinct biosynthetic pathways play in various aspects of leaf morphogenesis. Notably, mutants within the *YUCCA* gene family exhibit the most extensive and pleiotropic defects directly linked to leaf morphogenesis. Higher-order *yuc* mutants display not only significant differences in leaf size but also profound abnormalities in leaf curvature (epinasty/rolling) and even in the initiation of leaf development. This pronounced phenotype highlights the YUCCA-mediated auxin biosynthesis pathway as a central regulatory node that is critically required for the integrated control of leaf morphogenesis.

Among the four sub-routes, the IPyA pathway was the first to be identified and has been discovered to be highly conserved across terrestrial plants [53,77,78,79]. This pathway primarily consists of two steps: firstly, TRP is converted into the intermediate product IPyA by TRP aminotransferase (TAA/TAR); subsequently, IPyA is catalytically transformed into IAA by YUCCA enzymes. Recent studies have demonstrated that phosphorylation of the threonine residue at position 101 on TAA1, in conjunction with the coenzyme pyridoxal phosphate (PLP), functions as a regulatory switch for auxin synthesis [80]. Developmental changes associated with auxin biosynthesis have been observed in various mutants of *YUCCA* genes (Table 1), all of which exhibit a reduction in IAA content, irrespective of whether they are single, double, or multiple mutants. The cumulative evidence suggests that the *YUCCA* genes regulate multiple aspects of plant growth and development, including the formation of floral organs and vascular tissues, through the production of auxin characterized by precise temporal and spatial patterns.

The IAOx pathway is initiated by the cytochrome P450 enzymes CYP79B2 and CYP79B3, which convert TRP into IAOx. These enzymes have so far been conclusively identified only in *Brassicaceae* species, such as *Arabidopsis*, *Brassica napus*, and *Sinapis alba* [81,82]. Consequently, the IAOx-dependent route is regarded as a lineage-specific sub-pathway within the *Brassicaceae* family. In *Arabidopsis*, it represents a crucial metabolic branch point, channeling intermediates toward both auxin biosynthesis and the production of defense-related secondary metabolites, such as camalexin and indole glucosinolates [61,83]. It was commonly believed that in *Arabidopsis*, IAOx can be transformed into a variety of distinct products, including IAN and IG, which are ultimately converted into IAA by nitrilases (NITs). The enzymes CYP71A and CYP83B1 are responsible for the conversion of IAOx to IAN and IG, respectively [56,57,59,60]. However, this established downstream route has been fundamentally challenged by recent genetic evidence (Figure 2). High-order mutants that knock out all putative IAOx pathway genes in the three selected multigenic families—CYP71A, NIT, and AMI—which have been previously proposed to catalyze the conversion of IAN, IAM, or IAOx into IAA, retained their ability to produce IAA derived from IAOx and exhibited no apparent morphological defects [84]. This compelling genetic data indicates that these previously considered key gene families are dispensable for IAOx-mediated auxin biosynthesis in *Arabidopsis*. Consequently, the critical enzymatic steps converting IAOx to IAA in plants remain unidentified, pointing to the existence of a novel, yet-to-be-characterized enzyme or an alternative metabolic route. This revelation underscores the dynamic nature of auxin biosynthesis research and highlights a major unresolved question in the field.

The research indicates that cell-free extracts obtained from various plant species, including *Arabidopsis*, cauliflower, maize, potato, sunflower, tobacco, tomato, and white mustard, have the capability to convert TRP into IAM. This finding suggests that the IAM sub-pathway involved in IAA biosynthesis is widely distributed among plants [85]. It is speculated that IAM may be transformed by TRP with the catalysis of tryptophan-2-monooxygenase, which is encoded by genes homologous to the bacterial *aux1* gene. However, as of now, no relevant candidate gene has been identified in plants. In *Arabidopsis*, AtAMI1 and IAMHs have been identified as the primary enzymes responsible for converting IAM into IAA [74,75,76]. Furthermore, it has been reported that the levels of IAM showed a moderate restoration in the *cyp79b2 cyp79b3* double mutant when subjected to IAOx treatment, indicating that IAM production also involves the IAOx intermediate [61]. The IAM sub-route have been less studied than the IPyA and IAOx pathways. Given that the plant enzymes may exhibit considerable divergence from their bacterial counterparts, corresponding proteins might not be easily identifiable through homology searches. Therefore, comprehensive research is necessary to identify the enzymes associated with the IAM sub-route of IAA biosynthesis in plants.

The TAM sub-route represents one of the lesser-known pathways in IAA biosynthesis, involving the decarboxylation of TRP by Tryptophan Decarboxylase (TDC), a cytosolic enzyme that catalyzes the conversion of TRP into TAM. Although TDC has been isolated from several species, it remains unidentified in *Arabidopsis* [86,87,88,89]. The overexpression of the *TDC* gene from *C. roseus* in tobacco has resulted in the accumulation of significantly elevated levels of TAM, while IAA levels remained unchanged. It has been proposed that YUCCA enzymes catalyze a rate-limiting step within the TAM sub-route, specifically the N-hydroxylation of TAM to produce N-Hydroxytryptamine (NHT). The assertion that NHT is an in vitro product generated by YUCCA enzymes utilizing TAM as a substrate was based on gas chromatography-mass spectrometry (GC-MS) analyses conducted without authentic NHT serving as a control [90]. Subsequent investigations indicated that the spectra of authentic NHT were inconsistent with those of the previously reported compound [91]. Consequently, the role of YUCCA enzymes in TAM-dependent IAA biosynthesis has been called into question, and no definitive conclusions have been reached thus far.

Therefore, auxin biosynthesis in plants is primarily regulated through four distinct pathways, each involving specific enzymatic components. Among these, the YUCCA family of flavin monooxygenases has emerged as a key player, crucially regulating leaf development by precisely modulating local auxin accumulation through its spatiotemporally controlled expression [92]. In *Arabidopsis*, *YUCCA* genes respond to the adaxial-abaxial juxtaposition in leaves and are essential for leaf margin formation [93]. This localized expression generates discrete auxin maxima that coordinate cell proliferation and differentiation during blade outgrowth. Higher-order mutants of the *YUCCA* family display severe defects in leaf initiation, expansion, and patterning, underscoring the importance of spatially regulated auxin synthesis in translating developmental cues into leaf morphology. Thus, the YUCCA-mediated auxin biosynthesis module serves as a central integrator of positional signals and growth outputs during leaf development.

## 4. Auxin Transport and Distribution

The vascular system serves as a vital conduit for the long-distance transport of signaling molecules, such as hormones, which play a crucial role in coordinating the life activities of the entire plant. In this system, the two conducting tissues, xylem and phloem, run parallel to one another throughout the plant structure. Xylem vessels function similarly to drinking straws by harnessing the transpiration stream to transport water and dissolved nutrients from the roots to the shoots. Conversely, phloem functions as a living intracellular transport route that facilitates a more direct and precise regulation of signal transmission to target tissues [94,95].

PAT, which refers to the active transport of auxin between cells, underpins the chemiosmotic model. Specifically, the disparity in lipophilicity between auxin located in the apoplast and that residing in the cytoplasm establishes the foundation for auxin accumulation within the cytoplasmic milieu [8]. In the slightly acidic extracellular environment of the apoplast, with a pH of approximately 5.5, IAA exists predominantly in two forms: a membrane-impermeable form (IAA^−^) and a membrane-permeable form (IAAH), which facilitates IAA diffusion. Conversely, within the cytoplasm, characterized by a neutral pH, IAA primarily adopts its IAA^−^ form. Therefore, effective intercellular transport of IAA necessitates specific carriers embedded within the cell membrane. These carriers encompass AUXIN/LIKE-AUX proteins (AUX1/LAX) and P-glycoproteins (PGP) involved in auxin influx, along with PIN proteins and ABCB proteins responsible for auxin efflux [96,97].

In *Arabidopsis*, the *AUX1/LAX* gene family comprises four members: *AUX1*, *LAX1*, *LAX2*, and *LAX3*. The proteins encoded by these genes feature multiple transmembrane (TM) domains and exhibit similarities to amino acid transporters. *aux1* mutants display a phenotype characterized by auxin-resistant root growth and a complete loss of root gravitropic curvature [96,98]. Additionally, it has been reported that in *lax3* mutants, the number of emerged lateral roots decreases by approximately 40%, a reduction comparable to that observed in *aux1*. Moreover, the *aux1 lax3* double mutant displayed a markedly more severe phenotype with respect to lateral root development [99]. Genetic evidence indicates that *LAX2* is instrumental in regulating vascular development, while *AUX1*, *LAX1*, and *LAX2* collectively govern leaf phyllotactic patterning in a redundant manner [100,101]. These findings underscore the crucial role played by the *AUX1/LAX* gene family in auxin transport.

PIN proteins are essential components of the PAT mechanism, playing a crucial role in directing auxin flow within plant tissues and organs. This class of transmembrane proteins consists of eight members in *Arabidopsis*, which can be categorized into two subfamilies based on the presence or absence of a central hydrophilic domain. The larger PIN1 subfamily includes PIN1, PIN2, PIN3, PIN4, and PIN7, while the smaller PIN5 subfamily encompasses PIN5, PIN6, and PIN8 [102,103]. In the *pin1* mutant, the development of lateral organs in the inflorescence meristem is nearly completely inhibited. In comparison to the inflorescence meristem, phenotypic alterations in leaves are significantly less pronounced during both the cotyledon and vegetative growth stages. However, *pin1 pin4* double mutants exhibit severe defects affecting both the inflorescence meristem and leaf phenotypes [104]. The *pin2* mutants display root agravitropism, suggesting that accurate localization of PIN2 to the plasma membrane as well as its intracellular distribution pattern are essential for directional root growth [105]. The nutrient components present in the culture medium can reverse the non-gravitropic root phenotype of the *pin2* mutant, suggesting that this phenotype is conditional and sensitive to nutrient availability. Furthermore, ectopic expression of *PIN1* in the epidermis of the *pin2* mutant is associated with a restoration of gravitropic responsiveness [106].

The ABCB proteins exhibit two essential functional domains: the transmembrane domain (TMD), which functions as the site for substrate recognition and translocation across the membrane, and the nucleotide-binding domain (NBD), which is responsible for ATP binding and hydrolysis. Some members of the ABCB family, specifically those categorized as half-size ABCBs, possess one TMD and one NBD. In contrast, full-size ABCBs are characterized by two TMDs and two NBDs. These structural variations not only enhance their capacity to interact with a variety of compounds but also improve the transport efficiency of different ABCB proteins [107]. Currently, the transport activities of ABCB1, 4, 6, 11, 14, 15, 16, 17, 18, 19, 20, 21, and 22 have been confirmed in *Arabidopsis*. Furthermore, a conserved D/E-P motif has been identified as critical for their auxin transport activity [108,109,110]. Given the widespread prevalence and significant impact of functional redundancy, elucidating the role of ABCB-mediated auxin transport in developmental processes presents considerable challenges. This complexity is further supported by the increasingly severe phenotypes observed in double and higher-order mutants [108,110]. Moreover, it appears that ABCB transporters exhibit selectivity towards aromatic and aliphatic organic acids, a specificity that is notably enhanced when ABCBs are present alongside PINs and Twisted Dwarf 1 (TWD1) [109,111]. Further research is essential to ascertain the auxin transport properties of these ABCBs, potentially through biochemical assays and heterologous systems.

The root has been regarded as an exemplary model organ for investigating polar auxin transport due to its distinct structural polarity. Genetic evidence demonstrates that in mutants of auxin transporters, phenotypic defects are observed not only in root gravitropism but also frequently manifest prominently in leaf morphology. This suggests that the mechanism of polar auxin transport exhibits both universality and organ-specificity across various plant tissues. Therefore, by integrating classical findings from root studies with emerging insights from leaf research, we aim to provide a more comprehensive understanding of the mechanisms underlying polar auxin transport.

## 5. Auxin Signaling

Auxin initiates both rapid and slow cellular responses, forming a sophisticated and dynamic regulatory system in which auxin acts as a principal regulator, promptly and precisely directing plant development and environmental adaptation. The rapid responses occur within a brief timeframe, typically lasting less than one minute. These responses include cell membrane depolarization, oscillations of calcium ions, fluxes of hydrogen ions, protoplast swelling, reorganization of the cytoskeleton, and the regulation of endocytic trafficking involving auxin transporters [112,113]. In contrast, the slow response unfolds gradually over a period ranging from hours to days. This process is characterized by persistent changes in gene expression and protein synthesis that ultimately influence developmental processes through the regulation of cell growth, division, and differentiation. The swift advancement in molecular biology and biochemistry has led to the establishment of the well-recognized “central dogma of auxin signaling”, which contains a double-negative logic model and auxin activates transcription by degrading repressors of gene expression [114].

**Figure 3:**
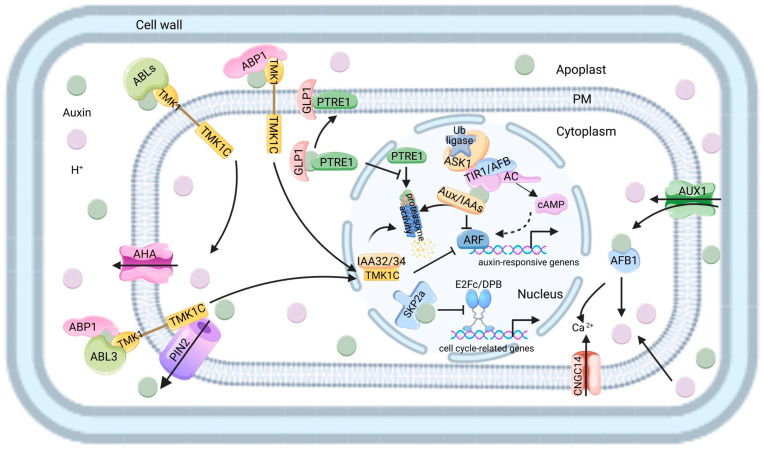
Auxin signaling pathways in *Arabidopsis*. The ABLs and ABP1 proteins physically interact with TMK1, forming a complex on the PM to sense auxin, which facilitates the interaction between TMK1 and PIN2, resulting in the phosphorylation of PIN2 and the asymmetric auxin flow. High concentration of auxin facilitates the cleavage of the TMK1 C-terminal domain (TMK1C). TMK1C then enters the nucleus, where it inhibits ARF-mediated gene expression by phosphorylating and stabilizing IAA proteins. In the nucleus, auxin binds to TIR1/AFBs via its auxiliary LRR domain while its F-box domain interacts with SCF through ASK1 protein, which subsequently mediates the degradation of Aux/IAA, thereby releasing the ARF to regulate the expression of down-stream genes. TIR1/AFB auxin receptors possess AC activity, thereby generating cAMP upon auxin binding. Local production of cAMP within the Aux/IAA-ARF regulatory niche circumvents auxin perception, promotes the degradation of Aux/IAAs, and activates ARF-mediated transcription. Auxin also binds to nucleus SKP2a, facilitating its interaction with cell cycle-related proteins E2Fc and DPB, which subsequently triggers the degradation of these proteins, thereby activating the expression of cell cycle-related genes. Auxin also enhances the interaction between GLP1 and PTRE1, facilitating PTRE1 retention at the plasma membrane. This redistribution of PTRE1 reduces nuclear 26S proteasome activity, leading to decreased degradation of Aux/IAA proteins. Auxin enters the plant cell through AUX1. In the cytoplasm, AFB1 triggers a rapid auxin response that relies on CNGC14-mediated Ca^2+^ signaling. PM, plasma membrane. Dashed arrows denote processes that have yet to be clearly elucidated.

The TIR1/AFBs-mediated transcriptional regulation is known as the classical auxin signaling pathway. In this pathway, auxin acts as a “molecular glue”, facilitating the interaction between the TIR1/AFB receptor and the Auxin/Indole-3-Acetic Acids (Aux/IAAs) transcriptional repressor. This interaction subsequently triggers the polyubiquitination and ensuing proteasomal degradation of the Aux/IAA protein, thereby alleviating the inhibitory effect exerted by the Aux/IAA repressor on *ARF* transcription factors. Ultimately, this process promotes the expression of auxin-responsive genes [115]. Recent discoveries [116,117,118] have revealed that TIR1/AFB auxin receptors possess adenylate cyclase (AC) activity, thereby generating cAMP upon auxin binding. Transgenic plants abolishing the AC activity of TIR1 demonstrate that the localized production of cAMP near the Aux/IAA-ARF complex, facilitated by unrelated AC enzymes, can circumvent the requirement for auxin perception and is sufficient to trigger ARF-mediated transcription. Without TIR1 AC activity, the auxin-induced degradation of Aux/IAAs is insufficient to mediate the transcriptional response to auxin. Although abolishing TIR1 AC activity does not interfere with the auxin-mediated degradation of Aux/IAAs, it renders TIR1 non-functional in facilitating transcriptional reprogramming and regulating auxin-dependent developmental processes, including shoot and root growth, root hair development, and lateral root formation. This groundbreaking discovery unveils a parallel signaling axis, wherein cAMP—generated as a direct consequence of auxin perception—functions as a positive co-signal to enhance *ARF* transcriptional activity. This finding fundamentally challenges the traditional model of canonical auxin signaling that has been established for the past two decades (Figure 3).

Relevant literature indicates that the diversity and complexity of the regulatory roles of auxin in plant development are achieved through the combination of multiple Aux/IAA-ARF transcriptional regulatory routes, highlighting the pivotal role of Aux/IAA in the classical nuclear auxin pathway [119]. The *Aux/IAA* gene family is extensive, with 29 members identified in *Arabidopsis*. Numerous studies have demonstrated that, compared to wild-type plants, single, double, and even higher-order mutants of *Aux/IAAs* typically exhibit no significant phenotypic differences or only minor defects, emphasizing the functional redundancy inherent within this gene family. The sextuple mutant (*iaa1 iaa2 iaa3 iaa4 iaa7 iaa16*) exhibits a more pronounced shade avoidance response, while the triple mutant comprising *iaa2*, *iaa7*, and *iaa16* also demonstrates a distinct phenotype reminiscent of shade avoidance. Notably, the expression levels of the *IAA2*, *IAA7*, and *IAA16* showed a significantly greater fold increase in response to shade avoidance compared to other *Aux/IAA* members [120]. The functional landscape of the *Aux/IAA* family, marked by significant genetic redundancy where individual members play critical roles based on their spatiotemporal expression patterns, needs re-evaluation in light of the newly discovered cAMP signaling branch. The cAMP model offers an alternative explanation: when the canonical degradation pathway is genetically compromised, the parallel cAMP-mediated activation can maintain substantial signaling output, providing functional compensation and resulting in a less severe phenotype than complete pathway loss. This shift suggests that the true null phenotype for integrated TIR1/AFB signaling—incorporating both degradation and cAMP pathways—is likely more severe than previously indicated by single or double mutant analyses, necessitating a reassessment of the genetic factors underlying the remarkable stability of the auxin signaling system.

Transmembrane kinases (TMKs) represents a class of receptor-like kinase proteins with leucine-rich repeats (LRR) in the extracellular domain and has long been recognized as a key factor in cell surface auxin signal transduction [3,11,121]. Notably, the TMK1 loss-of-function mutant does not exhibit any growth or developmental defects when compared to the wild type. However, both the *tmk1 tmk4* double mutant and the *tmk1 tmk3 tmk4* triple mutant display significantly reduced leaf sizes, primarily due to a decrease in cell number without any corresponding increase in cell size [11,122]. It is reported that TMK4 is involved in the auxin-mediated regulation of phosphorylation at the T101 residue of TAA1 proteins [54]. This indicates that auxin may facilitate a feedback mechanism that regulates its own biosynthesis, thereby sustaining the local auxin concentration required for specific developmental outcomes. TMK1 inhibits the phosphatase activity of ABA INSENSITIVE 2 (ABI2) by directly phosphorylating the highly conserved threonine 321 (T321) residue within ABI2 proteins. This phosphorylation state plays a critical role in mediating both auxin and ABA responses [123]. Within one minute of auxin treatment in *Arabidopsis* protoplasts, TMK proteins demonstrate an enhanced interaction with H^+^-ATPase (AHA), leading to the phosphorylation of the penultimate threonine residue within AHA [124].

Historically, the role of the extracellular auxin receptor has been debated. Although ABP1 was considered a candidate, genetic studies show that loss-of-function *abp1* mutants do not display significant developmental phenotypes, questioning its essentiality in various contexts [125,126]. ABP1 is part of the cupin superfamily and primarily localized to the endoplasmic reticulum, with limited presence at the cell surface, leaving the main apoplastic receptor unidentified. Recently, two apoplast-localized auxin-binding members of the GLP family, ABL1 and ABL2, were identified as direct auxin-dependent partners of TMK receptor kinases’ extracellular domains [11]. The *abl1/2* double mutant shows distinct morphological defects, while the *abp1;abl1/2* triple mutant presents even more severe phenotypes. This indicates that ABL1 and ABL2 likely act as primary apoplastic auxin receptors that functionally compensate for ABP1. Current evidence highlights ABL1/2 as key mediators of extracellular auxin perception related to TMK signaling, while ABP1’s role maybe context-specific and less essential in many developmental processes. This updated framework is illustrated in Figure 3.

Auxin enhances the interaction between GLP1 (also referred to as ABL1) and Proteasome regulator 1 (PTRE1), thereby facilitating the retention of PTRE1 at the plasma membrane. The redistribution of PTRE1 leads to a reduction in nuclear 26S proteasome activity, which subsequently results in diminished degradation of Aux/IAA proteins and altered Aux/IAA homeostasis [127]. This chain of events ultimately leads to the modulation of auxin-mediated transcriptional regulation. Auxin also rapidly triggers phosphorylation, leading to the stabilization of PIN2, through the cell-surface ABP1-TMK1 receptor module. Upon gravitropism stimulation, the initial auxin asymmetry activates the autophosphorylation of the TMK1 kinase. This, in turn, facilitates the interaction between TMK1 and PIN2, resulting in the phosphorylation of PIN2. Consequently, this stabilizes PIN2 at the lower root side, reinforcing the asymmetric auxin flow, which is essential for root bending. Additionally, upstream of TMK1 in this regulatory pathway, the root-expressed auxin receptor ABL3 functions redundantly alongside ABP1 [12]. Probing the auxin-GLP-TMK interface offers a compelling framework to explore the yet-uncharacterized developmental functions of GLPs. This line of investigation is poised to bridge the gap between extracellular hormone perception and intracellular signaling cascades, potentially unveiling novel regulatory modules that coordinate plant growth and patterning.

The rapid response of roots to auxin has been confirmed to depend on AFB1, a member of the TIR1/AFB auxin receptor family, which was previously believed to be exclusively involved in transcriptional responses [128,129]. Control plants began to exhibit gravity-induced bending within 2 to 4 min following gravitational stimulation. In contrast, the roots of the *afb1–3* single mutant displayed a delay of approximately 10 min—a response akin to that observed in roots lacking the CNGC14 calcium channel. This suggests that AFB1 may possess unique molecular characteristics that account for its specific functional role in the prompt inhibition of root growth by auxin [129,130]. Both nuclear AFB1 and cytoplasmic AFB1 are capable of inhibiting the canonical auxin signaling pathway [131]. In the case of nuclear AFB1, this protein may exert a dominant negative effect similar to that of TIR1; however, the precise mechanism by which cytoplasmic AFB1 inhibits the canonical pathway remains unclear. The key insight from these studies is the mechanistic link between auxin perception at the plasma membrane and cytosolic Ca^2+^ signaling activation. This cascade—from signal perception to biomechanical change—represents a fundamental mechanism for converting auxin gradients into coordinated tissue expansion. Although characterized in root tips, this logic is universally applicable. In leaf development, analogous mechanisms are likely responsible for driving cell expansion during blade outgrowth and for the fine-tuning of pavement cell shape.

There are six members of *Arabidopsis* Aux/IAA protein family (IAA20, IAA30, IAA31, IAA32, IAA33, and IAA34) that lack domain II, which is responsible for ubiquitination and subsequently the TIR1-mediated degradation of Aux/IAAs [132]. Under high concentrations of auxin, the cleaved TMK1C fragment can phosphorylate IAA32 and IAA34 in the nucleus, thereby enhancing their stability and regulating ARF-mediated gene transcription [3,133]. Additionally, IAA33 is also stabilized by auxin through MITOGEN-ACTIVATED PROTEIN KINASE 14 (MPK14), which subsequently allows it to compete with the canonical AUX/IAA repressor IAA5 for binding to *ARF10/16*. This competition protects *ARF10/16* from inhibition by IAA5, ultimately facilitating the transcription of auxin-related genes [134]. The precise mechanism by which auxin activates MAPK14 remains unclear; however, it is reasonable to speculate that this activation may occur via a pathway analogous to TMK1.

Furthermore, it has been demonstrated that *Arabidopsis* S-Phase Kinase-Associated Protein 2a (SKP2a), an F-box protein responsible for regulating the proteolysis of cell cycle transcription factors, is involved in auxin signaling. SKP serves as a crucial component of the SCF E3 ubiquitin ligase complex, which consists of SKP, Cullin, and F-box proteins. This complex significantly influences the stability and functionality of several key proteins associated with the cell cycle [135]. Auxin has been shown to enhance the interaction between SKP2A and the E2FC/DPB heterodimer, subsequently mediating the degradation of this protein complex. This process promotes the auxin-induced expression of genes related to the cell cycle [136,137]. These results demonstrate that SKP2A serves as a crucial connector between auxin and cell division.

In summary, auxin plays a pivotal role in regulating plant growth and development through various pathways. These pathways not only establish the groundwork for potential interactions among these mechanisms but also enhance the specificity of auxin responses. Consequently, a comprehensive analysis of such interactions is essential for a thorough understanding of the multifaceted functions of auxin in plant biology.

## 6. The Roles of Auxin in Leaf Morphogenesis

Leaf development is a well-organized and intricate process that involves the integration of various phytohormones, and this is also applicable to plant responses to stress [21,138,139]. Several classical phytohormones have been extensively studied, including auxin, cytokinins (CKs), gibberellins (GAs), brassinosteroids (BRs), strigolactones (SLs), abscisic acid (ABA), jasmonic acid (JA), and salicylic acid (SA). However, the key regulatory nodes mediating interactions between auxin and other hormones remain insufficiently characterized, and their spatiotemporal dynamics across various leaf developmental stages are yet to be systematically mapped. During the initiation of leaf primordia, auxin, GA and CKs act in a coordinated yet often antagonistic manner to balance meristem maintenance and organ differentiation. High accumulation of auxin in the shoot apical meristem (SAM) triggers the expression of *Class I KNOX* (*KNOXⅠ*) genes, which are instrumental in promoting leaf primordium initiation. Concurrently, *KNOXⅠ* stimulates CK biosynthesis to preserve SAM pluripotency and inhibits GA signaling by repressing *GA20ox* expression. This intricate regulatory network balances meristem activity with organ differentiation [140,141]. In the establishment of leaf polarity, *ARF* transcription factors serve as critical molecular hubs that integrate auxin and BR signaling networks by activating the expression of the key BR biosynthesis gene *DWARF4* (*DWF4*), thereby initiating a multi-layered regulatory circuit essential for precise adaxial-abaxial patterning [139,140]. In the morphogenesis stage, where final leaf size, shape, and margin patterning are determined, auxin synergizes with CKs and BRs to promote cell expansion and proliferation, whereas GAs, SLs, and JA often modulate or inhibit these processes [138,142]. Cell-cycle regulators, such as *cyclins B* (*CYCB*) and *D* (*CYCD*), may serve as potential integration nodes. BRs promote leaf growth by sustaining the expression of *CYCB* and facilitating cell-cycle progression. In contrast, JA inhibits leaf expansion by downregulating *CYCB*, thereby restricting cell division [139]. Auxin further contributes to the regulation of the cell cycle by indirectly activating *CYCD3*, which promotes the G1/S transition and coordinates with other hormones to drive leaf morphogenesis [140]. CUC2-PIN1-auxin genetic module plays a crucial role in the leaf margin formation, and CUC2 was found to limit growth rather by cell cycle inhibition than by cell size control [143].

Due to their sessile nature, plants inevitably encounter various biotic and abiotic stresses, prompting extensive research into the cross-regulation of phytohormones under stress conditions. While auxin cooperates with other hormones to regulate diverse developmental processes—such as root growth and flowering time—studies on leaf development under stress have largely focused on stress-induced leaf senescence [138,144]. The evidence we have gathered suggests that *ARF* transcription factors may act as key nodes mediating auxin-ABA crosstalk during leaf morphogenesis under stresses. This regulatory network involves miRNA-mediated layers: *miR160* targets specific *ARFs*, which in turn influence leaf development through downstream miRNAs essential for sequential developmental processes. Concurrently, *miR165/166* target *HD-ZIP III* transcription factors, contributing to drought tolerance by modulating genes involved in ABA biosynthesis, transport, and signaling. The interaction between *miR160* and *miR165/166* is further coordinated through a feedback loop linking *HD-ZIP IIIs* and *ARFs*, thereby integrating auxin and ABA pathways to fine-tune leaf development and stress adaptation [145]. Under conditions of low red and far-red light, the auxin-mediated induction of *Cytokinin Oxidase 6* (*AtCKX6*) facilitates the degradation of CKs, consequently leading to a reduction in cell proliferation within developing leaf primordia [146]. This regulatory mechanism exemplifies how variations in light quality dynamically influence auxin-cytokinin interactions, thereby fine-tuning the processes of leaf initiation and growth. Future research should clarify how phytohormones interact to regulate leaf morphogenesis under environmental stress, revealing the spatiotemporal dynamics of these interactions. Additionally, it is crucial to understand how plants balance normal leaf development with morphological plasticity for adaptation, as this knowledge could inform breeding of stress-resilient crops.

While multiple phytohormones coordinate to regulate leaf development, auxin often serves as a central orchestrator in this network. Its role goes beyond simple hormone interaction; auxin uses precise spatial-temporal distribution and signaling pathways to control critical developmental events. To clarify how auxin independently influences leaf morphogenesis, the following section examines its mechanisms demonstrating how localized auxin dynamics lead to organized growth and patterning.

### 6.1. Auxin in Leaf Development Initiation

Leaf development initiates from the SAM, which consists of embryonic cell populations organized into tunica and corpus layers. The SAM can be divided into three layers: L1, L2, and L3 [147]. Among these, the L1 layer serves as the promeristem layer that differentiates into the epidermis. This region exhibits the highest concentration of auxin, which is mediated by the polar-localized PIN1 efflux transporter. The areas with elevated auxin levels correspond to the sites where leaf primordia initiate. The vegetative *pin1* mutants exhibit variable timing in leaf initiation and display notably distinct divergence angles, both of which can be restored through exogenous application of auxin [15]. The majority of *pin1pin4* double mutant seedlings exhibit only a single cotyledon, and their leaf area is significantly smaller than that of the *pin1* seedlings [104]. In the SAM, PIN1 transports auxin to the initial site of leaf primordium, where elevated auxin concentrations inhibit the expression of *KNOXⅠ* genes. Additionally, *ASYMMETRIC LEAVES1* (*AS1*)/*ROUGH SHEATH2* (*RS2*)/*PHANTASTICA* (*ARP*) transcription factors can further suppress *KNOXⅠ* gene expression by binding to their promoters through the AS1/AS2 protein complex [21,148]. *KNOXⅠ* genes are crucial for maintaining meristem function, and their misexpression can result in various phenotypic alterations, including lobed leaves, ectopic stipules, and the formation of meristems in inter-lobe regions [149]. The AUX1/LAX family, which serves as auxin influx carriers, has also been demonstrated to be involved in regulating both the localization and initiation of leaf primordia via stabilizing PIN1-mediated auxin patterning [16,21].

Beyond this fundamental auxin-KNOX regulatory module, *KNOXⅠ* genes additionally play a pivotal role in the emergence of primordia through hormonal interactions. They positively regulate CK biosynthesis via Isopentenyl transferase 7 (IPT7) while concurrently exerting a negative influence on GA signaling by repressing GA20ox [140]. This intricate modulation ultimately shapes the growth environment for nascent primordia.

**Figure 4:**
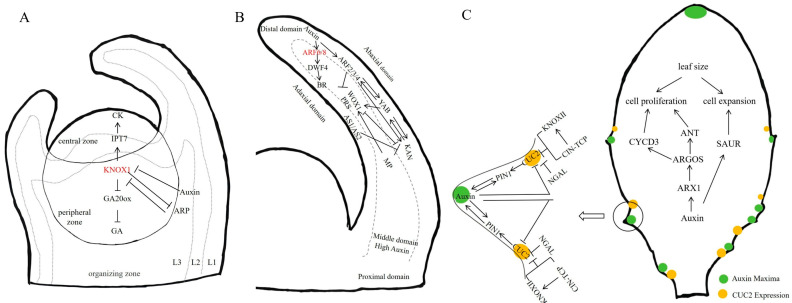
The roles of auxin in leaf morphogenesis. (**A**) Auxin in initiation of leaf primordium. Initiation of leaf primordium occurs in the SAM, which consists of three layers: L1, L2, and L3. Auxin accumulates on the flanks of the SAM through PIN1/AUX1-mediated transport, triggering primordia development. High auxin levels suppress KNOX1 expression. KNOX1 is essential for maintaining stem cell identity; it enhances CK levels and inhibits GA signaling via IPT7 and GA20oxidase, respectively. (**B**) Auxin in establishment of leaf polarity. Transcripts of AS1/AS2 accumulate in the adaxial domain of the leaf primordium, transcripts of ARF2/3/4, YAB and KAN accumulate in the abaxia domain, and WOX1/PRS are expressed in the intermediate domain of the leaf primordium. The transcription factors inhibit each other’s expression and control each other. High levels of auxin activates ARF, thereby facilitating the expression of YAB and KAN. Simultaneously, ARF2/3/4 and KAN were inhibited by AS1/AS2. KAN inhibited the expression of WOX1 and PRS, while WOX1 and PRS inhibited the expression of KAN. MP and off-axes enrichment of auxin together localized WOX1 and PRS expression in the intermediate domain. In addition, YAB promotes the expression of WOX1/PRS with KAN and ARF2/3/4. ARF6 and ARF8, induced by auxin, activate the expression of DWF4, thereby initiating the synthesis and signal transduction pathways of BRs. (**C**) For leaf shape formation, the feedback between auxin transport by PIN1 and up-the-gradient polar localization of PIN1 by auxin creates maxima in auxin concentration. This mechanism requires CUC2 for reorienting PINs. Auxin inhibits CUC2, stabilizing auxin maxima. The protrusions and indentations of serrations reflect areas of high auxin and CUC2 concentrations, respectively. Furthermore, the combination of CIN-TCP and KNOXII exerts a partial repressive effect on CUC2. For leaf size formation, Auxin induces the expression of ARGOS, which sustains the activity of the transcription factor ANT and a key cell cycle regulators, notably CYCD3, to promote cell proliferation, thereby increasing cell number. Auxin promotes cell expansion mainly via SAUR genes, which activate H^+^-ATPases in the plasma membrane. This process acidifies the apoplast, activates cell wall-loosening enzymes, and facilitates turgor-driven cell growth, ultimately determining leaf size. Note: → denotes positive regulation; T-shaped arrows indicate negative regulation.

In summary, leaf primordium initiation is controlled by PIN1-mediated auxin transport, establishing local auxin maxima at specific sites in the SAM. These auxin peaks suppress the expression of *KNOXI* genes, promoting the transition from indeterminate to determinate leaf fate. AUX1/LAX carriers stabilize the distribution of auxin, while *ARP* transcription factors further inhibit *KNOXIs* by binding to their respective promoters (Figure 4A).

### 6.2. Auxin in the Establishment of Leaf Polarity

Once the leaf primordium emerges at the periphery of the meristem, three axes that govern the polarized growth of leaf morphogenesis are already established: the adaxial-abaxial axis, which defines the front-back orientation of the leaf; the proximal-distal axis, indicating its base-tip orientation; and the medio-lateral axis, representing the primary vein-edge orientation of the leaf. The establishment of adaxial-abaxial polarity is of great significance in determining leaf polarity and thickness. Prior to the emergence of the basal swelling in the leaf primordium, the positional information encoded by the ASYMMETRIC LEAVES2-KANADI1 (AS2-KAN1) prepattern transforms the initially non-polar distribution of auxin within the leaf primordium into a polar configuration through an *ARF*-dependent auxin signal transduction pathway. Consequently, this process leads to the establishment of adaxial-abaxial polarity [17]. The *KAN* genes encode proteins that contain a GARP domain and are predominantly expressed in the abaxial domain. The triple mutant of *kan1kan2kan3* results in leaves displaying adaxial characteristics; conversely, the overexpression of individual *KAN* genes promotes abaxialization [150,151]. There exists a partial overlap between auxin and the downstream response factor *MONOPTEROS* (*MP*), leading to a pronounced auxin signal in the intermediate region situated between the adaxial and abaxial axes. The expression of *MP* on the adaxial side directly facilitates the accumulation of WOX1 and PRESSED FLOWER (PRS), both of which are critical determinants for establishing the intermediate zone essential for lateral blade expansion [21,22]. *ARF2*, *ARF3*, and *ARF4* are specifically expressed on the abaxial side of leaves and play a role in inhibiting the expression of *WOX1* and *PRS* within that region. The leaf blades produced by *arf3arf4* mutant exhibits outgrowths on their abaxial surface, a phenotype that closely resembles that of *kan* mutants [152].

The synergistic action of *MP* along with *ARF2*, *ARF3*, and *ARF4* facilitates the targeted expression of *WOX1* and *PRS* in the middle leaf domain, thereby facilitating leaf unfolding [18,140]. The YABBY (YAB) proteins have been shown to play a crucial role in establishing adaxial-abaxial polarity in leaves and are essential for defining the marginal regions during leaf development. The four members of the *YAB* gene family are specifically expressed on the abaxial side and within the marginal domain of leaf primordia, with their expression being regulated by *KAN* as well as *ARF2/3/4*. Notably, the loss-of-function mutant that lacks all four *YAB* genes exhibits reduced lamina growth; however, they display only limited defects in polarity [151,153].

The elongation of leaves is regulated by the proximal-distal axes, which are modulated by auxin. *ARF6* and *ARF8* activate the expression of *DWF4*, a gene that encodes a crucial enzyme involved in BR synthesis, thereby initiating the biosynthesis and signaling pathways associated with BRs. Subsequently, BRs facilitate the demethylation of cell wall pectin, resulting in isotropic in-plane loosening of the cell wall. Through its influence on BR biosynthesis, auxin affects cell wall mechanics and directs growth orientation at the cellular level, ultimately leading to diverse leaf shapes and governing the proximal-distal development of leaf reproductive structures [19].

The width of the leaf is influenced by the medio-lateral axis, within which *WOXs* serve as the most crucial regulatory factor. The expression of *WOX1* and *WOX3* genes is repressed by *KAN* and activated by *MP*. In the *kan1kan2* double mutant, both *WOX1* and *WOX3* are expressed in the abaxial domain [154,155]. Under the influence of *MP*, along with auxin enriched in the abaxial region, the expression of *WOX1* and *WOX3* is activated in both marginal and middle domains. Conversely, *ARF2/3/4*, which are expressed abaxially, effectively suppress the expression of *WOX1* and *WOX3* [20]. Plants expressing a dominant-negative form of *MP* exhibit narrow leaves, a phenotype that closely resembles that of the *wox1wox3* double mutant. In triple mutant lacking functional copies of *ARF2*, *ARF3*, and *ARF4*, *WOX1* and *WOX3* are ectopically expressed within the abaxial domain of leaf primordia [18]. Recently, it has been reported that loss of *WOX5* in the *wox1wox3* mutants leads to an additional reduction in leaf size and exacerbates the narrow leaf phenotype. This finding suggests that *WOX5* functions redundantly with both *WOX1* and *WOX3* in regulating leaf growth [23]. Additionally, in the region of leaf margins, *WOXs* exhibit the ability to regulate the expression of *YUCCA*, a key enzyme involved in auxin synthesis. Thus, the WOX-auxin regulatory module coordinates growth along both the proximo-distal and medio-lateral axes of the leaf, influencing its overall morphology.

To summarize, the establishment of leaf polarity is fundamentally governed by auxin signaling, in which key auxin biosynthesis enzymes, transport proteins, and transcription factors involved in signal transduction play essential roles (Figure 4B). The AS2-KAN1 prepattern translates positional information into a polarized distribution of auxin, which initiates the adaxial-abaxial specification. The transport of auxin, facilitated by PIN1 and proteins from the AUX1/LAX family, further refines this polarity. Additionally, auxin perception mediated through *ARF* transcription factors governs downstream gene expression. Furthermore, the WOX-YUCCA regulatory module integrates spatial patterning with localized auxin biosynthesis.

### 6.3. Auxin in Leaf Size and Shape Development

Following the establishment of leaf polarity, the leaf blade undergoes processes of cell proliferation and expansion, ultimately achieving its final size and shape [156]. In the early stages, a majority of cells within the primordia continuously divide, leading to a rapid increase in cell number; in contrast, cell size remains relatively stable during this phase. Subsequently, a transition occurs wherein most cell division ceases and cell expansion becomes the predominant process, culminating in significant cell enlargement. In the simplest scenario, the size of mature leaves is determined by both the cell number and cell size [157].

Auxin plays a crucial role in stimulating both cell proliferation and expansion, while also acting as a signaling molecule during cell division to regulate the final size of plant organs. It enhances the expression of the *AUXIN REGULATED GENE INVOLVED IN ORGAN SIZE* (*ARGOS*), along with a series of downstream genes, such as *AINTEGUMENTA* (*ANT*), *SMALL AUXIN UP RNA* (*SAUR*), which are essential regulators of plant cell expansion and proliferation [158,159,160]. Transgenic plants that express either sense or antisense *ARGOS* cDNA exhibit enlarged or reduced aerial organs, respectively, and the variation in organ size can be primarily attributed to changes in cell number and the duration of organ growth. In plants overexpressing *ARGOS*, sustained expression of *ANT* is noted in fully expanded leaves; however, this organ enlargement can be inhibited by loss-of-function mutations in *ANT*. This finding demonstrates that *ANT* operates downstream of *ARGOS* to regulate organ size [159]. The expression of *CycD3;1*, a D-type cyclin that acts as a regulator of the cell cycle and is also regulated by auxin, was found to be prolonged in leaf explants overexpressing either *ARGOS* or *ANT*. The overexpression of *CycD3;1* leads to the hyperproliferation of leaf cells; specifically, the epidermis consists of a significant number of small, incompletely differentiated polygonal cells [161]. The *auxin-resistant1* (*axr1*) mutant exhibits significantly smaller leaves, which can be attributed to a reduction in cell number rather than cell size [162]. The induction of *ARGOS* by auxin is reduced or entirely inhibited in the axr1 mutant; however, the overexpression of *ARGOS* partially restores organ development in axr1. These findings collectively suggest that *ARGOS* functions downstream of *AXR1* to regulate cell proliferation and organ growth through ANT during organogenesis [159]. *SAUR* genes have been extensively utilized as auxin-inducible reporters and certain *SAUR* genes have also been identified as being highly expressed in tissues undergoing differential cell expansion, particularly during tropic growth. Seedlings with gain-of-function and loss-of-function mutations in several *SAUR* genes exhibit increased and decreased leaf sizes, respectively [160]. Additionally, the functional loss of *ARF2* enhances cell proliferation, subsequently leading to an increase in leaf size [26].

*Arabidopsis thaliana* leaves are characterized by a spatulate shape with serrated margins. These serrations comprise small, regularly distributed protuberances interspersed with sinuses. In *Arabidopsis*, various components of the auxin signaling pathway significantly influence the formation of leaf margin serration. The key genetic factors implicated in the formation of these serrations are NAC-domain transcription factors, specifically CUC2 and CUC3. The CUC transcription factor is pivotal in the establishment and maintenance of meristem-organ boundary zones in *Arabidopsis* [25,163,164]. CUC1, together with CUC2 and CUC3, is crucial for defining the boundary between the SAM and emerging organ primordia. Mutants exhibiting a combinatorial loss of function demonstrate phenotypes such as cotyledon fusion and defects in SAM development. CUC2 plays a crucial role in the intracellular re-localization of the auxin efflux carrier PIN1 and is essential for the formation of auxin convergence points. Furthermore, auxin negatively regulates CUC2, thereby restricting its expression to regions located between outgrowths. Modifications to the CUC2-PIN1-auxin genetic module leads to distinct leaf phenotypes. The loss of either *PIN1* or *CUC2* leads to a complete absence of serrations, resulting in smooth leaf margins. Conversely, an increase in *CUC2* levels or disruptions in auxin signaling culminate in the development of highly lobed leaves [24,25]. The roles of *CUC* genes in leaf serration and leaflet development are partially mediated by their interaction with *KNOXI* genes. This mechanistic connection is crucial for the initiation of lateral organ primordia on the SAM, as the reciprocal activation between *KNOXI* and *CUC* genes delineates the boundary between the meristem and primordia, thereby facilitating proper outgrowth of lateral organs [165,166]. Transcription factors belonging to the *CINCINNATA-TEOSINTE BRANCHED1-CYCLOIDEA-PCF* (*CIN-TCP*) family also interact with *CUC* genes to regulate leaf margin growth. *CIN-TCP* genes negatively influence *CUCs* during leaf morphogenesis by promoting the expression of a range of *CUC* repressors, including *microRNA164*, *AS1*, and various genes associated with auxin functions [167]. Auxin regulates cell division by promoting the expression of *AS1* and *AS2*, thereby shaping leaf morphology. Both *as1* and *as2* single mutants exhibit a distinct bilateral asymmetrical growth pattern, resulting in the formation of lobes and lobular structures that originate from the petioles [168]. The *NGATHA-LIKE* (*NGAL*) transcription factors are also involved in the development of leaf margins by inhibiting the expression of *CUC2*. Notably, the *ngaltri* triple mutant, which exhibits a loss of function in NGAL1-3, presents an enhanced dentate leaf margin phenotype [169].

Briefly, auxin regulates leaf morphogenesis by coordinating cell proliferation and expansion through a hierarchical network (Figure 4C). Key downstream targets, such as ARGOS-ANT and SAUR pathways, convert auxin signals into specific cell cycle and growth outputs. Additionally, auxin influences leaf margin patterning through a feedback loop involving the CUC2-PIN1 module and interactions with KNOX, TCP, and NGAL transcription factors. These interconnected mechanisms collectively determine final leaf size and shape, demonstrating how auxin integrates growth and patterning during leaf development.

## 7. Conclusions and Perspectives

This review intentionally focuses on *Arabidopsis thaliana*, reflecting the current state of mechanistic research regarding auxin pathways. While auxin functions are broadly conserved across plant species, the gene families, expression patterns, and phenotypic outcomes discussed herein—particularly in biosynthesis, transport, and signaling—are most thoroughly understood within the *Arabidopsis* system. For mechanisms of leaf morphogenesis, our emphasis remains primarily on *Arabidopsis* to ensure a coherent and deeply mechanistic narrative.

Despite well-established core pathways of auxin biosynthesis, transport, and signaling, our understanding of auxin-mediated leaf development remains far from complete. Recent discoveries—such as refined models of IAOx-mediated auxin synthesis and novel auxin-GLP-TMK perception systems—highlight the dynamic and evolving nature of auxin biology while revealing substantial unexplored mechanistic domains. Critical gaps persist at multiple levels of regulation, limiting our ability to construct an integrated, spatially resolved network of auxin action in leaf morphogenesis.

At the genetic level, the functional redundancy and context-specific roles of auxin-related gene family members remain inadequately resolved. For instance, although *YUCCA* genes are critical for local auxin synthesis and the establishment of leaf polarity, it remains uncertain whether distinct homologous *YUCCA* genes specifically regulate localized auxin production during leaf polarity establishment through spatiotemporal expression patterns or variations in enzymatic activity beyond mere functional redundancy. At the interaction level, the molecular logic integrating auxin signaling with other developmental cues is still being mapped. For instance, how do tissue-specific *ARF* transcription factors, such as *ARF2/3/4* in abaxial domains, recruit transcriptional co-regulators to precisely regulate polarity establishment? How the spatiotemporal auxin biosynthesis fine-tuned during different stages of leaf development is also underexplored.

Research in model plants like *Arabidopsis* lays the groundwork for crop improvement by identifying functional genes and regulatory mechanisms that serve as key breeding targets. Advances in precision breeding, especially gene editing, now facilitate the targeted engineering of essential auxin pathway components to enhance crop architecture [170].

A primary goal in architecture is to develop crops with erect leaves and smaller auricles. This structure improves light penetration, allowing for higher planting density, maximizing photosynthetic efficiency and yield per area. Auxin transport plays a crucial role in determining these traits. For instance, multiplex CRISPR/Cas9 editing of *GmPIN1* in soybean modifies auxin distribution, fostering the development of erect leaves that are well-suited for dense planting [171]. Maize *pin* mutants exhibit reduced auricle size and altered leaf angles [172]. In contrast, rice *pin1* mutants experience drooping leaves accompanied by developmental complications [173,174]. Additionally, the knockout of *OsABCB24* in rice results in diminished IAA levels and increased leaf inclination [175]. Moreover, it has been reported that auxin response factors (ARFs) also contribute to the regulation of leaf angles; specifically, rice mutants *osarf4*/*osarf12* display enhanced leaf angles [176,177].

Beyond posture, leaf rolling represents another adaptive trait regulated by auxin. This characteristic enhances drought resilience by reducing water loss and optimizing light distribution within the canopy, thereby contributing to yield stability under stressful conditions. This morphological adaptation is closely linked to auxin signaling pathways, as demonstrated by tomato *SlARF4* mutants exhibiting upward-rolled leaves [178] and the curly leaf *GaIAA14* mutant in cotton [179]. Furthermore, disruptions in auxin biosynthesis also lead to changes in leaf shape, as evidenced by the rice mutant *Osyuc8*, which displays rolled leaves [180].

The manipulation of auxin pathways for crop improvement is promising but carries significant risks and trade-offs that require careful consideration. Firstly, pleiotropic effects pose a significant challenge, as auxin impacts various developmental processes beyond leaf architecture. Whether modifying key nodes unintentionally affects other traits, potentially compromising plant fitness or yield stability? Secondly, genetic redundancy in auxin-related gene families may cause compensatory mechanisms that obscure phenotypic effects or require multiplex editing, adding technical and regulatory complexity. Thirdly, achieving tissue- and stage-specific gene expression is crucial to prevent developmental abnormalities; constant overexpression or silencing often leads to serious morphological defects. Lastly, Environmental interactions can reveal unpredictable phenotypes in various genotypes or conditions, highlighting the importance of multi-environment testing and context-aware design. While auxin pathways provide great potential for customized leaf traits, their effective use necessitates precise spatial modulation and a systematic assessment of pleiotropy and trade-offs across various developmental stages and environments.

In summary, the morphogenetic principles that govern leaf development—mediated through spatially regulated auxin biosynthesis, polar transport, and signaling—are not merely descriptive; they possess direct functional implications for plant architecture and performance. By elucidating the mechanisms through which this conserved auxin network orchestrates leaf initiation, polarity, and final size, this review establishes a causal and mechanistic framework that lays the groundwork for precision breeding in crops. Importantly, key components within this framework are not merely theoretical; they have been validated as actionable targets in agricultural contexts. Manipulating these nodes has already resulted in agronomically significant phenotypes, including optimized leaf angles and improved canopy structures. Consequently, translating this integrated understanding—from foundational mechanisms observed in *Arabidopsis* to proof-of-concept applications in crops—into precision breeding pipelines represents the most direct and promising strategy for engineering next-generation crops with enhanced yield and sustainability.

## Figures and Tables

**Table 1:** Genes identified as being involved in TRP-dependent pathways of IAA synthesis in *Arabidopsis*.

Gene	Definition	Loss-of-Function Mutants	Phenotype	References
*TSA*	Trp synthase α	*trp3-1*	wider stomatal aperture; compressed root wave on tilted agar surfaces; sensitive to high light conditions; Trp auxotroph; high IAA and low Trp;	[4,48,50]
*TSB*	Trp synthase β	*trp2* *rtl2*	**reduced size of aerial organs, including leaves**, stems, flowers and siliques; **obviously small and chlorotic cotyledons with dark veins**; open-stomata and **dwarf**; compressed root wave phenotype on tilted agar surfaces; Trp auxotroph; high IAA and low Trp	[49,50,51,52]
*TAA/TAR*	Tryptophan Aminotransferase of *Arabidopsis*/Tryptophan Aminotransferase- Related	*taa1* *tar2*	*taa1*: **shorter petioles and larger leaf area than WT under shade;** disrupts root hair elongation under low phosphorus; root resistance to ethylene and N-1-Naphthylphtha-lamic acid(NPA); *tar2:* reduced lateral root number and density under low nitrate conditions	[53,54,55]
*CYP79B2/B3*	Cytochrome P450	*cyp79B2* *cyp79B3,* *cyp79B2 cyp79B3*	*cyp79B2 cyp79B3: ***shorter petioles and smaller leaves**; susceptible to *A. brassicicola;* strong accumulation of Trp; response to AgNO3 and UV irradiation;	[56,57,58]
*CYP83B1*	Cytochrome P450	*atr4* *sur2* *red1*	*sur2 and atr4: ***epinastic cotyledons**; elongated hypocotyl; increased lateral roots and adventitious roots originating from the hypocotyl; *red1:* **small cotyledons and elongated petioles**; reduced light responses to red light, including long hypocotyls	[59,60]
*NIT1/2/3*	Nitrilase	*nit1-3* *nit2* *nit3*	*nit1-3*: increased rosette leaf number and postponed flowing under short-day conditions; shorter hypocotyls under heat treatment; smaller root galls with a lower free IAA content; in response to *Plasmodiophora brassica*; insensitive to IAN; *nit2:* increased rosette leaf number and postponed flowing under short-day conditions; shorter hypocotyls under heat treatment; *nit3:* increased rosette leaf number and postponed flowing under short-day conditions	[61,62,63,64]
*AO1*	Aldehyde oxidase	*sur1*	**epinastic cotyledons**, elongated hypocotyls, excess adventitious and lateral roots, high IAA, high AO activity	[65,66]
*YUCCA*	Flavin monooxygenase-like enzyme	single mutant: *yuc1,* *yuc3,* *yuc4*; *yuc8* double mutants: *yuc1yuc4*, *yuc2yuc6*; triple mutants: *yuc1yuc2yuc6,* *yuc2yuc4yuc6*; quadruple mutants: *yuc1yuc2yuc4yuc6*, *yuc1yuc4yuc10yuc11*; quintuple mutant: *yuc3yuc5yuc7yuc8yuc9*	*yuc3*: shorter hypocotyls, defects in actin arrays; *yuc4*: reduced size of floral organs; *yuc8*: significantly defective gravitropic response; *yuc1yuc4*: **shorter rosette leaves**; decreased apical dominance; decreased numbers of stamen and carpel, completely sterile (defected in all four whorls of floral organs, and no functional reproductive organs); *yuc2yuc6*: **dark-green and shorter rosette leaves**; decreased apical dominance; shorter stamens, reduced fertility (late maturing anthers and no pollen produced); *yuc1yuc2yuc6* and *yuc2yuc4yuc6*: similar to *yuc2yuc6*; *yuc1yuc2yuc4* and *yuc1yuc4yuc6*: **curly leaves**; smaller statures and more extreme in decreased apical dominance than *yuc1yuc4*; floral defects in all four whorls, including fewer flowers and smaller flower structures than *yuc1yuc4*; *yuc1yuc2yuc4yuc6*: **dark-green, shorter and curly leaves**; stronger phenotypes than the triple mutants described above; *yuc1yuc4yuc10yuc11*: embryonic developmental defects; *yuc3yuc5yuc7yuc8yuc9*: short primary roots and had agravitropic root growth, decreased free IAA content	[67,68,69,70,71,72,73]
*IAMH*	Indole-3-acetamide hydrolase	*iamh1 iamh2*	No obvious developmental defects	[74]
*AMI1*	Indole-3-acetamide amidohydrolase	*ami1*	moderately repressed growth, increased abiotic stress sensitivity, high IAM and low IAA content, enhanced ABA accumulation	[75,76]

## Data Availability

The authors will make the raw data supporting the conclusions of this article accessible upon request.

## References

[1] Abel S., Theologis A. (2010). Odyssey of auxin. Cold Spring Harb. Perspect. Biol..

[2] Fattorini L., Veloccia A., Della Rovere F., D’Angeli S., Falasca G., Altamura M.M. (2017). Indole-3-butyric acid promotes adventitious rooting in *Arabidopsis thaliana* thin cell layers by conversion into indole-3-acetic acid and stimulation of anthranilate synthase activity. BMC Plant Biol..

[3] Cao M., Chen R., Li P., Yu Y., Zheng R., Ge D., Zheng W., Wang X., Gu Y., Gelová Z. (2019). TMK1-mediated auxin signalling regulates differential growth of the apical hook. Nature.

[4] Normanly J., Cohen J.D., Fink G.R. (1993). *Arabidopsis thaliana* auxotrophs reveal a tryptophan-independent biosynthetic pathway for indole-3-acetic acid. Proc. Natl. Acad. Sci. USA.

[5] Wang B., Chu J., Yu T., Xu Q., Sun X., Yuan J., Xiong G., Wang G., Wang Y., Li J. (2015). Tryptophan-independent auxin biosynthesis contributes to early embryogenesis in *Arabidopsis*. Proc. Natl. Acad. Sci. USA.

[6] Di D.W., Zhang C., Luo P., An C.W., Guo G.Q. (2016). The biosynthesis of auxin: How many paths truly lead to IAA?. Plant Growth Regul..

[7] Tang W., Yu Y., Xu T. (2025). The interplay between extracellular and intracellular auxin signaling in plants. J. Genet. Genom..

[8] Gomes G.L.B., Scortecci K.C. (2021). Auxin and its role in plant development: Structure, signalling, regulation and response mechanisms. Plant Biol..

[9] Prát T., Hajný J., Grunewald W., Vasileva M., Molnár G., Tejos R., Schmid M., Sauer M., Friml J. (2018). WRKY23 is a component of the transcriptional network mediating auxin feedback on PIN polarity. PLoS. Genet..

[10] Mazur E., Kulik I., Hajný J., Friml J. (2020). Auxin canalization and vascular tissue formation by TIR1/AFB-mediated auxin signaling in *Arabidopsis*. New Phytol..

[11] Yu Y., Tang W., Lin W., Li W., Zhou X., Li Y., Chen R., Zheng R., Qin G., Cao W. (2023). ABLs and TMKs are co-receptors for extracellular auxin. Cell.

[12] Rodriguez L., Fiedler L., Zou M., Giannini C., Monzer A., Vladimirtsev D., Randuch M., Yu Y., Gelová Z., Verstraeten I. (2025). ABP1/ABL3-TMK1 cell-surface auxin signaling targets PIN2-mediated auxin fluxes for root gravitropism. Cell.

[13] Friml J. (2022). Fourteen Stations of Auxin. Cold Spring Harb. Perspect. Biol..

[14] Lee Z.H., Hirakawa T., Yamaguchi N., Ito T. (2019). The roles of plant hormones and their interactions with regulatory genes in determining meristem activity. Int. J. Mol. Sci..

[15] Reinhardt D., Pesce E.R., Stieger P., Mandel T., Baltensperger K., Bennett M., Traas J., Friml J., Kuhlemeier C. (2003). Regulation of phyllotaxis by polar auxin transport. Nature.

[16] Robert H.S., Grunewald W., Sauer M., Cannoot B., Soriano M., Swarup R., Weijers D., Bennett M., Boutilier K., Friml J. (2015). Plant embryogenesis requires AUX/LAX-mediated auxin influx. Development.

[17] Burian A., Paszkiewicz G., Nguyen K.T., Meda S., Raczyńska-Szajgin M., Timmermans M.C.P. (2022). Specification of leaf dorsiventrality via a prepatterned binary readout of a uniform auxin input. Nat. Plants.

[18] Guan C., Wu B., Yu T., Wang Q., Krogan N.T., Liu X., Jiao Y. (2017). Spatial auxin signaling controls leaf flattening in *Arabidopsis*. Curr. Biol..

[19] Xiong Y., Wu B., Du F., Guo X., Tian C., Hu J., Lü S., Long M., Zhang L., Wang Y. (2021). A crosstalk between auxin and brassinosteroid regulates leaf shape by modulating growth anisotropy. Mol. Plant.

[20] Qi J., Wang Y., Yu T., Cunha A., Wu B., Vernoux T., Meyerowitz E., Jiao Y. (2014). Auxin depletion from leaf primordia contributes to organ patterning. Proc. Natl. Acad. Sci. USA.

[21] Wang H., Kong F., Zhou C. (2021). From genes to networks: The genetic control of leaf development. J. Integr. Plant Biol..

[22] Kalve S., De Vos D., Beemster G.T. (2014). Leaf development: A cellular perspective. Front. Plant Sci..

[23] Zhang Z., Runions A., Mentink R.A., Kierzkowski D., Karady M., Hashemi B., Huijser P., Strauss S., Gan X., Ljung K. (2020). A WOX/Auxin biosynthesis module controls growth to shape leaf form. Curr. Biol..

[24] Nikovics K., Blein T., Peaucelle A., Ishida T., Morin H., Aida M., Laufs P. (2006). The balance between the MIR164A and CUC2 genes controls leaf margin serration in *Arabidopsis*. Plant Cell.

[25] Bilsborough G.D., Runions A., Barkoulas M., Jenkins H.W., Hasson A., Galinha C., Laufs P., Hay A., Prusinkiewicz P., Tsiantis M. (2011). Model for the regulation of *Arabidopsis thaliana* leaf margin development. Proc. Natl. Acad. Sci. USA.

[26] Okushima Y., Mitina I., Quach H.L., Theologis A. (2005). AUXIN RESPONSE FACTOR 2 (ARF2): A pleiotropic developmental regulator. Plant J..

[27] Damodaran S., Strader L.C. (2019). Indole 3-butyric acid metabolism and transport in *Arabidopsis thaliana*. Front. Plant Sci..

[28] Du M., Spalding E.P., Gray W.M. (2020). Rapid Auxin-Mediated Cell Expansion. Annu. Rev. Plant Biol..

[29] Uzunova V.V., Quareshy M., Del Genio C.I., Napier R.M. (2016). Tomographic docking suggests the mechanism of auxin receptor TIR1 selectivity. Open Biol..

[30] Kreiser M., Giblin C., Murphy R., Fiesel P., Braun L., Johnson G., Wyse D., Cohen J.D. (2016). Conversion of Indole-3-Butyric Acid to Indole-3-Acetic Acid in shoot tissue of Hazelnut (*Corylus*) and Elm (*Ulmus*). J. Plant Growth Regul..

[31] Jawahir V., Zolman B.K. (2021). Long chain acyl CoA synthetase 4 catalyzes the first step in peroxisomal indole-3-butyric acid to IAA conversion. Plant Physiol..

[32] Aihebaier S., Muhammad T., Wei A., Mamat A., Abuduaini M., Pataer P., Yigaimu A., Yimit A. (2019). Membrane-Protected molecularly imprinted polymer for the microextraction of Indole-3-butyric acid in Mung Bean Sprouts. Am. Chem. Soc. Omega.

[33] Li Y.H., Mo Y.W., Wang S.B., Zhang Z. (2020). Auxin efflux carriers, MiPINs, are involved in adventitious root formation of mango cotyledon segments. Plant Physiol. Biochem..

[34] Velada I., Cardoso H., Porfirio S., Peixe A. (2020). Expression profile of PIN-formed auxin efflux carrier genes during IBA-induced in vitro adventitious rooting in *Olea europaea* L.. Plants.

[35] Choi M., Sathasivam R., Nguyen B.V., Park N.I., Woo S.H., Park S.U. (2021). Expression analysis of phenylpropanoid pathway genes and metabolomic analysis of phenylpropanoid compounds in adventitious, hairy, and seedling roots of Tartary Buckwheat. Plants.

[36] Hsieh Y.H., Wei Y.H., Lo J.C., Pan H.Y., Yang S.Y. (2022). Arbuscular mycorrhizal symbiosis enhances tomato lateral root formation by modulating CEP2 peptide expression. New Phytol..

[37] Justamante M.S., Mhimdi M., Molina-Pérez M., Albacete A., Moreno M.Á., Mataix I., Pérez-Pérez J.M. (2022). Effects of auxin (Indole-3-butyric Acid) on adventitious root formation in peach-based prunus rootstocks. Plants.

[38] Chen W., Shan W., Niu T., Ye T., Sun Q., Zhang J. (2023). Insight into regulation of adventitious root formation by arbuscular mycorrhizal fungus and exogenous auxin in tea plant (*Camellia sinensis* L.) cuttings. Front. Plant Sci..

[39] Trunjaruen A., Luecha P., Taratima W. (2023). The optimization of medium conditions and auxins in the induction of adventitious roots of Pokeweed (*Phytolacca americana* L.) and their phytochemical constituents. Scientifica.

[40] Damodaran S., Strader L.C. (2024). Factors governing cellular reprogramming competence in *Arabidopsis* adventitious root formation. Dev. Cell.

[41] Lam H.K., Ross J.J., McAdam E.L., McAdam S.A. (2016). The single evolutionary origin of chlorinated auxin provides a phylogenetically informative trait in the *Fabaceae*. Plant Signal. Behav..

[42] Jayasinghege C.P.A., Ozga J.A., Nadeau C.D., Kaur H., Reinecke D.M. (2019). TIR1 auxin receptors are implicated in the differential response to 4-Cl-IAA and IAA in developing pea fruit. J. Exp. Bot..

[43] Ozga J.A., Jayasinghege C.P.A., Kaur H., Gao L., Nadeau C.D., Reinecke D.M. (2022). Auxin receptors as integrators of developmental and hormonal signals during reproductive development in pea. J. Exp. Bot..

[44] Sugawara S., Mashiguchi K., Tanaka K., Hishiyama S., Sakai T., Hanada K., Kinoshita-Tsujimura K., Yu H., Dai X., Takebayashi Y. (2015). Distinct characteristics of Indole-3-Acetic Acid and phenylacetic acid, two common auxins in plants. Plant Cell Physiol..

[45] Perez V.C., Zhao H., Lin M., Kim J. (2023). Occurrence, function, and biosynthesis of the natural auxin phenylacetic acid (PAA) in plants. Plants.

[46] Wright A.D., Sampson M.B., Neuffer M.G., Michalczuk L., Slovin J.P., Cohen J.D. (1991). Indole-3-Acetic acid biosynthesis in the mutant Maize orange pericarp, a Tryptophan auxotroph. Science.

[47] Niyogi K.K., Fink G.R. (1992). Two anthranilate synthase genes in *Arabidopsis*: Defense-related regulation of the tryptophan pathway. Plant Cell.

[48] Radwanski E.R., Barczak A.J., Last R.L. (1996). Characterization of tryptophan synthase alpha subunit mutants of *Arabidopsis thaliana*. Mol. Gen. Genet..

[49] Ursache R., Miyashima S., Chen Q., Vatén A., Nakajima K., Carlsbecker A., Zhao Y., Helariutta Y., Dettmer J. (2014). Tryptophan-dependent auxin biosynthesis is required for HD-ZIP III-mediated xylem patterning. Development.

[50] Soda M.N., Hayashi Y., Takahashi K., Kinoshita T. (2022). Tryptophan synthase ß subunit 1 affects stomatal phenotypes in *Arabidopsis thaliana*. Front. Plant Sci..

[51] Ouyang J., Shao X., Li J. (2000). Indole-3-glycerol phosphate, a branchpoint of indole-3-acetic acid biosynthesis from the tryptophan biosynthetic pathway in *Arabidopsis thaliana*. Plant J..

[52] Jing Y., Cui D., Bao F., Hu Z., Qin Z., Hu Y. (2009). Tryptophan deficiency affects organ growth by retarding cell expansion in *Arabidopsis*. Plant J..

[53] Won C., Shen X., Mashiguchi K., Zheng Z., Dai X., Cheng Y., Kasahara H., Kamiya Y., Chory J., Zhao Y. (2011). Conversion of tryptophan to indole-3-acetic acid by TRYPTOPHAN AMINOTRANSFERASES OF *ARABIDOPSIS* and YUCCAs in *Arabidopsis*. Proc. Natl. Acad. Sci. USA.

[54] Ma W., Li J., Qu B., He X., Zhao X., Li B., Fu X., Tong Y. (2014). Auxin biosynthetic gene TAR2 is involved in low nitrogen-mediated reprogramming of root architecture in *Arabidopsis*. Plant J..

[55] Bhosale R., Giri J., Pandey B.K., Giehl R.F.H., Hartmann A., Traini R., Truskina J., Leftley N., Hanlon M., Swarup K. (2018). A mechanistic framework for auxin dependent *Arabidopsis* root hair elongation to low external phosphate. Nat. Commun..

[56] Nafisi M., Goregaoker S., Botanga C.J., Glawischnig E., Olsen C.E., Halkier B.A., Glazebrook J. (2007). *Arabidopsis cytochrome* P450 monooxygenase 71A13 catalyzes the conversion of indole-3-acetaldoxime in camalexin synthesis. Plant Cell.

[57] Müller T.M., Böttcher C., Morbitzer R., Götz C.C., Lehmann J., Lahaye T., Glawischnig E. (2015). TRANSCRIPTION ACTIVATOR-LIKE EFFECTOR NUCLEASE-mediated generation and metabolic analysis of Camalexin-deficient cyp71a12 cyp71a13 double knockout lines. Plant Physiol..

[58] Zhao Y., Hull A.K., Gupta N.R., Goss K.A., Alonso J., Ecker J.R., Normanly J., Chory J., Celenza J.L. (2002). Trp-dependent auxin biosynthesis in *Arabidopsis*: Involvement of cytochrome P450s CYP79B2 and CYP79B3. Genes Dev..

[59] Barlier I., Kowalczyk M., Marchant A., Ljung K., Bhalerao R., Bennett M., Sandberg G., Bellini C. (2000). The SUR2 gene of *Arabidopsis thaliana* encodes the cytochrome P450 CYP83B1, a modulator of auxin homeostasis. Proc. Natl. Acad. Sci. USA.

[60] Hoecker U., Toledo-Ortiz G., Bender J., Quail P.H. (2004). The photomorphogenesis-related mutant red1 is defective in CYP83B1, a red light-induced gene encoding a cytochrome P450 required for normal auxin homeostasis. Planta.

[61] Sugawara S., Hishiyama S., Jikumaru Y., Hanada A., Nishimura T., Koshiba T., Zhao Y., Kamiya Y., Kasahara H. (2009). Biochemical analyses of indole-3-acetaldoxime-dependent auxin biosynthesis in *Arabidopsis*. Proc. Natl. Acad. Sci. USA.

[62] Grsic-Rausch S., Kobelt P., Siemens J.M., Bischoff M., Ludwig-Müller J. (2000). Expression and localization of nitrilase during symptom development of the clubroot disease in *Arabidopsis*. Plant Physiol..

[63] van der Woude L., Piotrowski M., Klaasse G., Paulus J.K., Krahn D., Ninck S., Kaschani F., Kaiser M., Novák O., Ljung K. (2021). The chemical compound ‘Heatin’ stimulates hypocotyl elongation and interferes with the *Arabidopsis* NIT1-subfamily of nitrilases. Plant J..

[64] Yang S., Zhang T., Wang Z., Zhao X., Li R., Li J. (2022). Nitrilases NIT1/2/3 positively regulate flowering by inhibiting MAF4 expression in *Arabidopsis*. Front. Plant Sci..

[65] Seo M., Akaba S., Oritani T., Delarue M., Bellini C., Caboche M., Koshiba T. (1998). Higher activity of an aldehyde oxidase in the auxin-overproducing superroot1 mutant of *Arabidopsis thaliana*. Plant Physiol..

[66] Sekimoto H., Seo M., Kawakami N., Komano T., Desloire S., Liotenberg S., Marion-Poll A., Caboche M., Kamiya Y., Koshiba T. (1998). Molecular cloning and characterization of aldehyde oxidases in *Arabidopsis thaliana*. Plant Cell Physiol..

[67] Cheng Y., Dai X., Zhao Y. (2006). Auxin biosynthesis by the YUCCA flavin monooxygenases controls the formation of floral organs and vascular tissues in *Arabidopsis*. Genes Dev..

[68] Chen Q., Dai X., De-Paoli H., Cheng Y., Takebayashi Y., Kasahara H., Kamiya Y., Zhao Y. (2014). Auxin overproduction in shoots cannot rescue auxin deficiencies in *Arabidopsis* roots. Plant Cell Physiol..

[69] Xu Y., Prunet N., Gan E.S., Wang Y., Stewart D., Wellmer F., Huang J., Yamaguchi N., Tatsumi Y., Kojima M. (2018). SUPERMAN regulates floral whorl boundaries through control of auxin biosynthesis. EMBO J..

[70] Luo W.G., Liang Q.W., Su Y., Huang C., Mo B.X., Yu Y., Xiao L.T. (2023). Auxin inhibits chlorophyll accumulation through ARF7-IAA14-mediated repression of chlorophyll biosynthesis genes in *Arabidopsis*. Front. Plant Sci..

[71] Yu H., Maoliniyazi M., Han X., Yang H., Zhang Z., Guo Y., Tang X., Li H., Cao Q., Wang S. (2025). YUCCA3 interacts with ADF4 to regulate *Arabidopsis* hypocotyl elongation by organizing actin arrays. Plant Physiol. Biochem..

[72] Liang Q., Lei J., Luo W., Wu S., Sun M., Deng D., Su Y., Xiao L. (2025). Filament elongation and pollen development regulated by auxin is crucial for male fertility in *Arabidopsis*. Plant Physiol. Biochem..

[73] Di D.W., Wu L., Zhang L., An C.W., Zhang T.Z., Luo P., Gao H.H., Kriechbaumer V., Guo G.Q. (2016). Functional roles of *Arabidopsis* CKRC2/YUCCA8 gene and the involvement of PIF4 in the regulation of auxin biosynthesis by cytokinin. Sci. Rep..

[74] Gao Y., Dai X., Aoi Y., Takebayashi Y., Yang L., Guo X., Zeng Q., Yu H., Kasahara H., Zhao Y. (2020). Two homologous INDOLE-3-ACETAMIDE (IAM) HYDROLASE genes are required for the auxin effects of IAM in *Arabidopsis*. J. Genet. Genom..

[75] Pérez-Alonso M.M., Ortiz-García P., Moya-Cuevas J., Lehmann T., Sánchez-Parra B., Björk R.G., Karim S., Amirjani M.R., Aronsson H., Wilkinson M.D. (2021). Endogenous indole-3-acetamide levels contribute to the crosstalk between auxin and abscisic acid, and trigger plant stress responses in *Arabidopsis*. J. Exp. Bot..

[76] Ortiz-García P., Pérez-Alonso M.M., González Ortega-Villaizán A., Sánchez-Parra B., Ludwig-Müller J., Wilkinson M.D., Pollmann S. (2022). The Indole-3-Acetamide-Induced *Arabidopsis* transcription factor MYB74 decreases plant growth and contributes to the control of osmotic stress responses. Front. Plant Sci..

[77] Phillips K.A., Skirpan A.L., Liu X., Christensen A., Slewinski T.L., Hudson C., Barazesh S., Cohen J.D., Malcomber S., McSteen P. (2011). Vanishing tassel2 encodes a grass-specific tryptophan aminotransferase required for vegetative and reproductive development in maize. Plant Cell.

[78] Yoshikawa T., Ito M., Sumikura T., Nakayama A., Nishimura T., Kitano H., Yamaguchi I., Koshiba T., Hibara K., Nagato Y. (2014). The rice FISH BONE gene encodes a tryptophan aminotransferase, which affects pleiotropic auxin-related processes. Plant J..

[79] Eklund D.M., Ishizaki K., Flores-Sandoval E., Kikuchi S., Takebayashi Y., Tsukamoto S., Hirakawa Y., Nonomura M., Kato H., Kouno M. (2015). Auxin produced by the Indole-3-pyruvic acid pathway regulates development and gemmae dormancy in the Liverwort *Marchantia polymorpha*. Plant Cell.

[80] Wang Q., Qin G., Cao M., Chen R., He Y., Yang L., Zeng Z., Yu Y., Gu Y., Xing W. (2020). A phosphorylation-based switch controls TAA1-mediated auxin biosynthesis in plants. Nat. Commun..

[81] Bak S., Nielsen H.L., Halkier B.A. (1998). The presence of CYP79 homologues in glucosinolate-producing plants shows evolutionary conservation of the enzymes in the conversion of amino acid to aldoxime in the biosynthesis of cyanogenic glucosides and glucosinolates. Plant Mol. Biol..

[82] Mano Y., Nemoto K. (2012). The pathway of auxin biosynthesis in plants. J. Exp. Bot..

[83] Cao X., Yang H., Shang C., Ma S., Liu L., Cheng J. (2019). The roles of auxin biosynthesis YUCCA gene family in plants. Int. J. Mol. Sci..

[84] Fenech M., Brumos J., Pěnčík A., Edwards B., Belcapo S., DeLacey J., Patel A., Kater M., Li X., Ljung K. (2025). The CYP71A, NIT, AMI, and IAMH gene families are dispensable for indole-3-acetaldoxime-mediated auxin biosynthesis in *Arabidopsis*. Plant Cell.

[85] Pollmann S., Düchting P., Weiler E.W. (2009). Tryptophan-dependent indole-3-acetic acid biosynthesis by ‘IAA-synthase’ proceeds via indole-3-acetamide. Phytochemistry.

[86] Songstad D.D., De Luca V., Brisson N., Kurz W.G., Nessler C.L. (1990). High levels of tryptamine accumulation in transgenic tobacco expressing tryptophan decarboxylase. Plant Physiol..

[87] López-Meyer M., Nessler C.L. (1997). Tryptophan decarboxylase is encoded by two autonomously regulated genes in *Camptotheca acuminata* which are differentially expressed during development and stress. Plant J..

[88] Yamazaki Y., Sudo H., Yamazaki M., Aimi N., Saito K. (2003). Camptothecin biosynthetic genes in hairy roots of *Ophiorrhiza pumila*: Cloning, characterization and differential expression in tissues and by stress compounds. Plant Cell Physiol..

[89] Kang S., Kang K., Lee K., Back K. (2007). Characterization of rice tryptophan decarboxylases and their direct involvement in serotonin biosynthesis in transgenic rice. Planta.

[90] Zhao Y., Christensen S.K., Fankhauser C., Cashman J.R., Cohen J.D., Weigel D., Chory J. (2001). A role for flavin monooxygenase-like enzymes in auxin biosynthesis. Science.

[91] Tivendale N.D., Davies N.W., Molesworth P.P., Davidson S.E., Smith J.A., Lowe E.K., Reid J.B., Ross J.J. (2010). Reassessing the role of N-hydroxytryptamine in auxin biosynthesis. Plant Physiol..

[92] Cheng Y., Dai X., Zhao Y. (2007). Auxin synthesized by the YUCCA flavin monooxygenases is essential for embryogenesis and leaf formation in *Arabidopsis*. Plant Cell.

[93] Wang W., Xu B., Wang H., Li J., Huang H., Xu L. (2011). YUCCA genes are expressed in response to leaf adaxial-abaxial juxtaposition and are required for leaf margin development. Plant Physiol..

[94] Aoki K., Suzui N., Fujimaki S., Dohmae N., Yonekura-Sakakibara K., Fujiwara T., Hayashi H., Yamaya T., Sakakibara H. (2005). Destination-selective long-distance movement of phloem proteins. Plant Cell.

[95] Zhang Y., Berman A., Shani E. (2023). Plant Hormone Transport and Localization: Signaling Molecules on the Move. Annu. Rev. Plant Biol..

[96] Swarup R., Bhosale R. (2019). Developmental roles of AUX1/LAX auxin influx carriers in plants. Front. Plant Sci..

[97] Mellor N.L., Voß U., Ware A., Janes G., Barrack D., Bishopp A., Bennett M.J., Geisler M., Wells D.M., Band L.R. (2022). Systems approaches reveal that ABCB and PIN proteins mediate co-dependent auxin efflux. Plant Cell.

[98] Bennett M.J., Marchant A., Green H.G., May S.T., Ward S.P., Millner P.A., Walker A.R., Schulz B., Feldmann K.A. (1996). *Arabidopsis* AUX1 gene: A permease-like regulator of root gravitropism. Science.

[99] Swarup K., Benková E., Swarup R., Casimiro I., Péret B., Yang Y., Parry G., Nielsen E., De Smet I., Vanneste S. (2008). The auxin influx carrier LAX3 promotes lateral root emergence. Nat. Cell Biol..

[100] Bainbridge K., Guyomarc’h S., Bayer E., Swarup R., Bennett M., Mandel T., Kuhlemeier C. (2008). Auxin influx carriers stabilize phyllotactic patterning. Genes Dev..

[101] Péret B., Swarup K., Ferguson A., Seth M., Yang Y., Dhondt S., James N., Casimiro I., Perry P., Syed A. (2012). AUX/LAX genes encode a family of auxin influx transporters that perform distinct functions during *Arabidopsis* development. Plant Cell.

[102] Simon S., Skupa P., Viaene T., Zwiewka M., Tejos R., Klíma P., Čarná M., Rolčík J., De Rycke R., Moreno I. (2016). PIN6 auxin transporter at endoplasmic reticulum and plasma membrane mediates auxin homeostasis and organogenesis in *Arabidopsis*. New Phytol..

[103] Zwiewka M., Bilanovičová V., Seifu Y.W., Nodzyński T. (2019). The nuts and bolts of PIN auxin efflux carriers. Front. Plant Sci..

[104] Guenot B., Bayer E., Kierzkowski D., Smith R.S., Mandel T., Žádníková P., Benková E., Kuhlemeier C. (2012). Pin1-independent leaf initiation in *Arabidopsis*. Plant Physiol..

[105] Peng Y., Ji K., Mao Y., Wang Y., Korbei B., Luschnig C., Shen J., Benková E., Friml J., Tan S. (2024). Polarly localized Bro1 domain proteins regulate PIN-FORMED abundance and root gravitropic growth in *Arabidopsis*. Commun. Biol..

[106] Thomas M., Soriano A., O’Connor C., Crabos A., Nacry P., Thompson M., Hrabak E., Divol F., Péret B. (2023). pin2 mutant agravitropic root phenotype is conditional and nutrient-sensitive. Plant Sci..

[107] Xu T., Dai N., Chen J., Nagawa S., Cao M., Li H., Zhou Z., Chen X., De Rycke R., Rakusová H. (2014). Cell surface ABP1-TMK auxin-sensing complex activates ROP GTPase signaling. Science.

[108] Chen J., Hu Y., Hao P., Tsering T., Xia J., Zhang Y., Roth O., Njo M.F., Sterck L., Hu Y. (2023). ABCB-mediated shootward auxin transport feeds into the root clock. EMBO Rep..

[109] Hao P., Xia J., Liu J., Di Donato M., Pakula K., Bailly A., Jasinski M., Geisler M. (2020). Auxin-transporting ABC transporters are defined by a conserved D/E-P motif regulated by a prolylisomerase. J. Biol. Chem..

[110] Jenness M.K., Tayengwa R., Bate G.A., Tapken W., Zhang Y., Pang C., Murphy A.S. (2022). Loss of multiple ABCB auxin transporters recapitulates the major twisted dwarf 1 phenotypes in *Arabidopsis thaliana*. Front. Plant Sci..

[111] Deslauriers S.D., Spalding E.P. (2021). Electrophysiological study of *Arabidopsis* ABCB4 and PIN2 auxin transporters: Evidence of auxin activation and interaction enhancing auxin selectivity. Plant Direct..

[112] Narasimhan M., Gallei M., Tan S., Johnson A., Verstraeten I., Li L., Rodriguez L., Han H., Himschoot E., Wang R. (2021). Systematic analysis of specific and nonspecific auxin effects on endocytosis and trafficking. Plant Physiol..

[113] Fiedler L., Friml J. (2023). Rapid auxin signaling: Unknowns old and new. Curr. Opin. Plant Biol..

[114] Morffy N., Strader L.C. (2022). Structural Aspects of Auxin Signaling. Cold Spring Harb. Perspect. Biol..

[115] Tan X., Calderon-Villalobos L.I., Sharon M., Zheng C., Robinson C.V., Estelle M., Zheng N. (2007). Mechanism of auxin perception by the TIR1 ubiquitin ligase. Nature.

[116] Qi L., Kwiatkowski M., Chen H., Hoermayer L., Sinclair S., Zou M., Del Genio C.I., Kubeš M.F., Napier R., Jaworski K. (2022). Adenylate cyclase activity of TIR1/AFB auxin receptors in plants. Nature.

[117] Chen H., Qi L., Zou M., Lu M., Kwiatkowski M., Pei Y., Jaworski K., Friml J. (2025). TIR1-produced cAMP as a second messenger in transcriptional auxin signalling. Nature.

[118] Vanneste S., Pei Y., Friml J. (2025). Mechanisms of auxin action in plant growth and development. Nat. Rev. Mol. Cell Biol..

[119] Jing H., Yang X., Emenecker R.J., Feng J., Zhang J., Figueiredo M.R.A., Chaisupa P., Wright R.C., Holehouse A.S., Strader L.C. (2023). Nitric oxide-mediated S-nitrosylation of IAA17 protein in intrinsically disordered region represses auxin signaling. J. Genet. Genom..

[120] Yang X., Ma Y., Chen J., Huang M., Qi M., Han N., Bian H., Qiu T., Yan Q., Wang J. (2024). Sextuple knockouts of a highly conserved and coexpressed AUXIN/INDOLE-3-ACETIC ACID gene set confer shade avoidance-like responses in *Arabidopsis*. Plant Cell Environ..

[121] Xu Y.X., Liu Y., Chen S.T., Li X.Q., Xu L.G., Qi Y.H., Jiang D.A., Jin S.H. (2014). The B subfamily of plant ATP binding cassette transporters and their roles in auxin transport. Biol. Plant..

[122] Huang R., Zheng R., He J., Zhou Z., Wang J., Xiong Y., Xu T. (2019). Noncanonical auxin signaling regulates cell division pattern during lateral root development. Proc. Natl. Acad. Sci. USA.

[123] Yang J., He H., He Y., Zheng Q., Li Q., Feng X., Wang P., Qin G., Gu Y., Wu P. (2021). TMK1-based auxin signaling regulates abscisic acid responses via phosphorylating ABI1/2 in *Arabidopsis*. Proc. Natl. Acad. Sci. USA.

[124] Lin W., Zhou X., Tang W., Takahashi K., Pan X., Dai J., Ren H., Zhu X., Pan S., Zheng H. (2021). TMK-based cell-surface auxin signalling activates cell-wall acidification. Nature.

[125] Gao Y., Zhang Y., Zhang D., Dai X., Estelle M., Zhao Y. (2015). Auxin binding protein 1 (ABP1) is not required for either auxin signaling or *Arabidopsis* development. Proc. Natl. Acad. Sci. USA.

[126] Napier R. (2021). The story of Auxin-Binding Protein 1 (ABP1). Cold Spring Harb. Perspect. Biol..

[127] Xu F., Yu Y., Guan B., Xu T., Xu Z., Xue H. (2025). Germin-like protein 1 interacts with proteasome regulator 1 to regulate auxin signaling by controlling Aux/IAA homeostasis. Cell Rep..

[128] Prigge M.J., Platre M., Kadakia N., Zhang Y., Greenham K., Szutu W., Pandey B.K., Bhosale R.A., Bennett M.J., Busch W. (2020). Genetic analysis of the *Arabidopsis* TIR1/AFB auxin receptors reveals both overlapping and specialized functions. eLife.

[129] Serre N.B.C., Kralík D., Yun P., Slouka Z., Shabala S., Fendrych M. (2021). AFB1 controls rapid auxin signalling through membrane depolarization in *Arabidopsis thaliana* root. Nat. Plants.

[130] Shih H.W., DePew C.L., Miller N.D., Monshausen G.B. (2015). The Cyclic Nucleotide-Gated Channel CNGC14 regulates root gravitropism in *Arabidopsis thaliana*. Curr. Biol..

[131] Dubey S.M., Han S., Stutzman N., Prigge M.J., Medvecká E., Platre M.P., Busch W., Fendrych M., Estelle M. (2023). The AFB1 auxin receptor controls the cytoplasmic auxin response pathway in *Arabidopsis thaliana*. Mol. Plant.

[132] Abel S., Oeller P.W., Theologis A. (1994). Early auxin-induced genes encode short-lived nuclear proteins. Proc. Natl. Acad. Sci. USA.

[133] Gu B., Dong H., Smith C., Cui G., Li Y., Bevan M.W. (2022). Modulation of receptor-like transmembrane kinase 1 nuclear localization by DA1 peptidases in *Arabidopsis*. Proc. Natl. Acad. Sci. USA.

[134] Lv B., Yu Q., Liu J., Wen X., Yan Z., Hu K., Li H., Kong X., Li C., Tian H. (2020). Non-canonical AUX/IAA protein IAA33 competes with canonical AUX/IAA repressor IAA5 to negatively regulate auxin signaling. EMBO J..

[135] del Pozo J.C., Boniotti M.B., Gutierrez C. (2002). *Arabidopsis* E2Fc functions in cell division and is degraded by the ubiquitin-SCF(AtSKP2) pathway in response to light. Plant Cell.

[136] del Pozo J.C., Diaz-Trivino S., Cisneros N., Gutierrez C. (2006). The balance between cell division and endoreplication depends on E2FC-DPB, transcription factors regulated by the ubiquitin-SCFSKP2A pathway in *Arabidopsis*. Plant Cell.

[137] Jurado S., Abraham Z., Manzano C., López-Torrejón G., Pacios L.F., Del Pozo J.C. (2010). The *Arabidopsis* cell cycle F-box protein SKP2A binds to auxin. Plant Cell.

[138] Zhao B., Liu Q., Wang B., Yuan F. (2021). Roles of phytohormones and their signaling pathways in leaf development and stress responses. J. Agric. Food Chem..

[139] Mazzoni-Putman S.M., Brumos J., Zhao C., Alonso J.M., Stepanova A.N. (2021). Auxin interactions with other hormones in plant development. Cold Spring Harb. Perspect. Biol..

[140] Lv Z., Zhao W., Kong S., Li L., Lin S. (2023). Overview of molecular mechanisms of plant leaf development: A systematic review. Front. Plant Sci..

[141] Hussain S., Nanda S., Zhang J., Rehmani M.I.A., Suleman M., Li G., Hou H. (2021). Auxin and Cytokinin interplay during leaf morphogenesis and phyllotaxy. Plants.

[142] Navarro-Cartagena S., Micol J.L. (2023). Is auxin enough? Cytokinins and margin patterning in simple leaves. Trends Plant Sci..

[143] Li X., Zheng Y., Xing Q., Ardiansyah R., Zhou H., Ali S., Jing T., Tian J., Song X.S., Li Y. (2020). Ectopic expression of the transcription factor CUC2 restricts growth by cell cycle inhibition in *Arabidopsis* leaves. Plant Signal. Behav..

[144] Gao J., Zhuang S., Zhang W. (2024). Advances in plant Auxin biology: Synthesis, metabolism, signaling, interaction with other hormones, and roles under abiotic stress. Plants.

[145] Yang T., Wang Y., Teotia S., Wang Z., Shi C., Sun H., Gu Y., Zhang Z., Tang G. (2019). The interaction between miR160 and miR165/166 in the control of leaf development and drought tolerance in *Arabidopsis*. Sci. Rep..

[146] Carabelli M., Possenti M., Sessa G., Ciolfi A., Sassi M., Morelli G., Ruberti I. (2007). Canopy shade causes a rapid and transient arrest in leaf development through auxin-induced cytokinin oxidase activity. Genes Dev..

[147] Kwiatkowska D. (2004). Structural integration at the shoot apical meristem: Models, measurements, and experiments. Am. J. Bot..

[148] Lodha M., Marco C.F., Timmermans M.C. (2013). The ASYMMETRIC LEAVES complex maintains repression of KNOX homeobox genes via direct recruitment of Polycomb-repressive complex2. Genes Dev..

[149] Ori N., Eshed Y., Chuck G., Bowman J.L., Hake S. (2000). Mechanisms that control knox gene expression in the *Arabidopsis* shoot. Development.

[150] Kerstetter R.A., Bollman K., Taylor R.A., Bomblies K., Poethig R.S. (2001). KANADI regulates organ polarity in *Arabidopsis*. Nature.

[151] Eshed Y., Izhaki A., Baum S.F., Floyd S.K., Bowman J.L. (2004). Asymmetric leaf development and blade expansion in *Arabidopsis* are mediated by KANADI and YABBY activities. Development.

[152] Pekker I., Alvarez J.P., Eshed Y. (2005). Auxin response factors mediate *Arabidopsis* organ asymmetry via modulation of KANADI activity. Plant Cell.

[153] Sarojam R., Sappl P.G., Goldshmidt A., Efroni I., Floyd S.K., Eshed Y., Bowman J.L. (2010). Differentiating *Arabidopsis* shoots from leaves by combined YABBY activities. Plant Cell.

[154] Nakayama N., Smith R.S., Mandel T., Robinson S., Kimura S., Boudaoud A., Kuhlemeier C. (2012). Mechanical regulation of auxin-mediated growth. Curr. Biol..

[155] Caggiano M.P., Yu X., Bhatia N., Larsson A., Ram H., Ohno C.K., Sappl P., Meyerowitz E.M., Jönsson H., Heisler M.G. (2017). Cell type boundaries organize plant development. eLife.

[156] Du F., Guan C., Jiao Y. (2018). Molecular mechanisms of leaf morphogenesis. Mol. Plant.

[157] Efroni I., Eshed Y., Lifschitz E. (2010). Morphogenesis of simple and compound leaves: A critical review. Plant Cell.

[158] Mizukami Y., Fischer R.L. (2000). Plant organ size control: AINTEGUMENTA regulates growth and cell numbers during organogenesis. Proc. Natl. Acad. Sci. USA.

[159] Hu Y., Xie Q., Chua N.H. (2003). The *Arabidopsis* auxin-inducible gene ARGOS controls lateral organ size. Plant Cell.

[160] Spartz A.K., Lee S.H., Wenger J.P., Gonzalez N., Itoh H., Inzé D., Peer W.A., Murphy A.S., Overvoorde P.J., Gray W.M. (2012). The SAUR19 subfamily of SMALL AUXIN UP RNA genes promote cell expansion. Plant J..

[161] Dewitte W., Riou-Khamlichi C., Scofield S., Healy J.M., Jacqmard A., Kilby N.J., Murray J.A. (2003). Altered cell cycle distribution, hyperplasia, and inhibited differentiation in *Arabidopsis* caused by the D-type cyclin CYCD3. Plant Cell.

[162] Esmon C.A., Tinsley A.G., Ljung K., Sandberg G., Hearne L.B., Liscum E. (2006). A gradient of auxin and auxin-dependent transcription precedes tropic growth responses. Proc. Natl. Acad. Sci. USA.

[163] Hu Z.L., Wilson-Sánchez D., Bhatia N., Rast-Somssich M.I., Wu A., Vlad D., McGuire L., Nikolov L.A., Laufs P., Gan X. (2024). A CUC1/auxin genetic module links cell polarity to patterned tissue growth and leaf shape diversity in crucifer plants. Proc. Natl. Acad. Sci. USA.

[164] Scofield S., Murison A., Jones A., Fozard J., Aida M., Band L.R., Bennett M., Murray J.A.H. (2018). Coordination of meristem and boundary functions by transcription factors in the SHOOT MERISTEMLESS regulatory network. Development.

[165] Spinelli S.V., Martin A.P., Viola I.L., Gonzalez D.H., Palatnik J.F. (2011). A mechanistic link between STM and CUC1 during *Arabidopsis* development. Plant Physiol..

[166] Balkunde R., Kitagawa M., Xu X.M., Wang J., Jackson D. (2017). SHOOT MERISTEMLESS trafficking controls axillary meristem formation, meristem size and organ boundaries in *Arabidopsis*. Plant J..

[167] Koyama T., Furutani M., Tasaka M., Ohme-Takagi M. (2007). TCP transcription factors control the morphology of shoot lateral organs via negative regulation of the expression of boundary-specific genes in *Arabidopsis*. Plant Cell.

[168] Semiarti E., Ueno Y., Tsukaya H., Iwakawa H., Machida C., Machida Y. (2001). The ASYMMETRIC LEAVES2 gene of *Arabidopsis thaliana* regulates formation of a symmetric lamina, establishment of venation and repression of meristem-related homeobox genes in leaves. Development.

[169] Shao J., Meng J., Wang F., Shou B., Chen Y., Xue H., Zhao J., Qi Y., An L., Yu F. (2020). NGATHA-LIKEs control leaf margin development by repressing CUP-SHAPED COTYLEDON2 transcription. Plant Physiol..

[170] Hou M., Zhang Y., Xu X., Ai H. (2025). Advances in auxin synthesis, transport, and signaling in rice: Implications for stress resilience and crop improvement. Front. Plant Sci..

[171] Zhang Z., Gao L., Ke M., Gao Z., Tu T., Huang L., Chen J., Guan Y., Huang X., Chen X. (2022). GmPIN1-mediated auxin asymmetry regulates leaf petiole angle and plant architecture in soybean. J. Integr. Plant Biol..

[172] Zhong Z., Yao M., Cao Y., Kong D., Wang B., Wang Y., Shen R., Wang H., Liu Q. (2025). LG1 promotes preligule band formation through directly activating ZmPIN1 genes in maize. J. Genet. Genom..

[173] Zhang Y., Han S., Lin Y., Qiao J., Han N., Li Y., Feng Y., Li D., Qi Y. (2023). Auxin transporter OsPIN1b, a novel regulator of leaf inclination in rice (*Oryza sativa* L.). Plants.

[174] Liu J., Shi X., Zhong T., Jie W., Xu R., Ding Y., Ding C. (2023). PINOID and PIN-FORMED paralogous genes are required for leaf morphogenesis in rice. Plant Cell Physiol..

[175] Yang Y., Tang Z., Zhang W.W., Huang X.Y., Zhao F.J. (2025). The chloroplast-localized ABC transporter OsABCB24 regulates aleurone cell size and grain nutritional quality in rice by modulating auxin homeostasis. J. Exp. Bot..

[176] Xian F., Liu S., Xie B., Huang J., Zhang Q., Xu Y., Zhang X., Lv C., Zhu L., Hu J. (2025). The auxin response factor OsARF12 modulates rice leaf angle via affecting shoot gravitropism. J. Genet. Genom..

[177] Qiao J., Zhang Y., Han S., Chang S., Gao Z., Qi Y., Qian Q. (2022). OsARF4 regulates leaf inclination via auxin and brassinosteroid pathways in rice. Front. Plant Sci..

[178] Liu X., Lin Y., Wu C., Yang Y., Su D., Xian Z., Zhu Y., Yu C., Hu G., Deng W. (2023). The SlARF4-SlHB8 regulatory module mediates leaf rolling in tomato. Plant Sci..

[179] Miao P., Zhang H., Xu Y., Zhang R., Hao Y., Song G., Liu J. (2025). A single-nucleotide mutation of G301A in GaIAA14 confers leaf curling in *Gossypium arboreum*. Front. Plant Sci..

[180] Zhou L., Chen S., Cai M., Cui S., Ren Y., Zhang X., Liu T., Zhou C., Jin X., Zhang L. (2023). ESCRT-III component OsSNF7.2 modulates leaf rolling by trafficking and endosomal degradation of auxin biosynthetic enzyme OsYUC8 in rice. J. Integr. Plant Biol..

